# Neurodegeneration and contralateral α-synuclein induction after intracerebral α-synuclein injections in the anterior olfactory nucleus of a Parkinson’s disease A53T mouse model

**DOI:** 10.1186/s40478-019-0713-7

**Published:** 2019-04-15

**Authors:** Alicia Flores-Cuadrado, Daniel Saiz-Sanchez, Alicia Mohedano-Moriano, Alino Martinez-Marcos, Isabel Ubeda-Bañon

**Affiliations:** 1 Neuroplasticity and Neurodegeneration Laboratory, CRIB, Ciudad Real Medical School, University of Castilla-La Mancha, Camino de Moledores s/n, Ciudad Real, 13071 Spain; 2 School of Occupational Therapy, Speech Therapy and Nursing, University of Castilla-La Mancha, Talavera de la Reina, Spain

**Keywords:** α-Synucleinopathy, Astroglia, Microglia, Neurodegeneration, Non-motor symptoms, Olfaction, Stereology

## Abstract

Parkinson’s disease is characterized by a proteinopathy that includes aggregates of α-synuclein. A recent hypothesis proposes a prion-like spreading mechanism for this α-synucleinopathy. Early neuropathological deposits occur, among others, in the anterior olfactory nucleus (AON). This study investigates the anterograde and/or retrograde transmissibility of exogenous α-synuclein inoculated in the right AON of the A53T model of Parkinson’s disease and wild-type mice as well as neuronal and glial involvement. Seven experimental groups were established: wild-type injected with tracers; A53T mice injected with either α-synuclein or saline 2 months beforehand; wild-type injected with either α-synuclein or saline 2 months beforehand; and wild-type injected with either α-synuclein or saline 4 months beforehand. Weight and behavioral changes were analyzed. Immunohistochemistry against α-synuclein, NeuN, Iba-1 and GFAP was performed. Volume and marker distributions in the olfactory bulb (OB), AON and piriform cortex were analyzed using unbiased stereology. The behavioral analyses reveal higher levels of hyperactivity in transgenic as compared to wild-type mice. Tract-tracing experiments show that the main contralateral afferent projections to the dorsal AON come from the AON and secondarily from the OB. In saline-injected transgenic animals, α-synuclein expression in the OB and the AON is higher in the left hemisphere than in the right hemisphere, which could be due to basal interhemispheric differences. α-synuclein injection could provoke a significant increase in the left hemisphere of the transgenic mice’s OB, compared to saline-injected animals. Neuronal loss was observed in saline-injected transgenic mice relative to the saline-injected wild-type group. There were no overall differences in neuron number following injection of α-synuclein into either wild-type or transgenic mice, however some neuron loss was apparent in specific regions of α-synuclein injected wild-types. Microglia labeling appeared to be correlated with surgery-induced inflammation. Astroglial labeling was higher in transgenic animals, which could be due to endogenous α-synucleinopathy. This study suggests α-synucleinopathy induction, via retrograde and contralateral projections, within the olfactory system of transgenic animals.

**Electronic supplementary material:**

The online version of this article (10.1186/s40478-019-0713-7) contains supplementary material, which is available to authorized users.

## Introduction

Parkinson’s disease (PD) is the second most prevalent neurodegenerative disease (affecting 2–3% of the population ≥ 65 years of age). It is mostly idiopathic and clinically diagnosed with the presence of bradykinesia in combination with rigidity and/or rest tremor [56, 59]. Motor symptoms are due to neuronal loss of dopaminergic cells in the substantia nigra pars compacta and the corresponding striatal denervation [39]. A prodromal period characterized by non-motor features, such as rapid eye movement sleep behavior disorder, constipation and hyposmia has been described [17, 70, 71]. PD is a synucleinopathy characterized by aggregates of α-synuclein and ubiquitin (Lewy bodies and neurites) which follows a progressive and predictable staging pattern [5], although the correlation between clinical manifestations and pathological deposits is far from straightforward [36]. Interestingly, Lewy pathology first appears in the dorsal motor nucleus of the vagus nerve and in the olfactory bulb (OB) [14, 15], particularly in the anterior olfactory nucleus (AON) [2, 4, 12, 72, 76, 77, 79].

The idea that proteinopathies associated with neurodegenerative diseases could spread in a prion-like manner, thus helping to explain their etiology, was proposed more than three decades ago [38, 60–62, 83]. Prion-like hypothesis is based on a conformational change from α-helix native α-synuclein into β-sheet form through a seeding mechanism and subsequent spreading process [29]. Unfolded α-synuclein monomers are able to turn into oligomers, and thereafter to amyloid fibrils. These fibrils give rise to Lewy bodies formation within neurons and glial cells [28, 42]. In the last decade this hypothesis has been supported by experimental data in PD [8, 74]. In vitro and in vivo data regarding neuronal α-synuclein spreading have been obtained in several species, and a number of mechanisms have been proposed for axonal [13] and transneuronal transport [18, 33, 74]. Further, it has been demonstrated in vitro that astrocytes can take up α-synuclein through an endocytic event and spread this α-synuclein to neurons, hence leading to neural death [10], although, in vivo, glial role on this process has been hardly investigated.

Neuroinflammation is a complex and dynamic response involved in PD. It has been suggested that microglia and astroglia have a major role in the onset, progression [27, 34] and degeneration associated to PD [7, 43]. The crosstalk between α-synuclein and glial cells is particularly interesting since α-synuclein is able to activate and direct microglia and its migration [40]. Also, microglia is responsible of clearing extracellular α-synuclein [44]. This microgliosis has been observed in PD post-mortem areas [19], including OB [41], in vivo, using PET imaging in prodromal (REM sleep disorder patients) and diagnosed PD patients [23, 75] and also in mouse models [43].

Not only microglia is relevant in PD, α-synuclein aggregates have been also reported in astrocytes [6]. A53T α-synuclein exclusively expressed in astrocytes, it is able to accelerate onset of movement disorders, disease progression and inflammatory response in mice [30]. Astrocytes establish communications with neurons, microglia and other astrocytes. Among their main roles are: homeostasis, injury repair and inhibition of microglial activation in healthy brain [32, 37]. Astrogliosis become active in diseased brain to protect neurons from oxidative stress and prevent excessive inflammation. In fact, some PD related genes are expressed in astrocytes and microglia [37]. There are multiple mechanisms by α-synuclein spreading as an intercellular transfer. α-Synuclein fibrils could transfer from neuron to neuron and from neuron to glial cells, even these cells are able to internalize them [1]. In fact, it has been suggested that glial cells have a dual role as collaborators in the α-synuclein spreading or/and its degradation [47].

Hodologically, several studies have demonstrated neural transmission of exogenous, intracerebrally injected α-synuclein in connected areas involved in PD: striatal injections and retrograde transport to the substantia nigra pars compacta [48]; nigral injections and anterograde and retrograde transport in the hippocampus, amygdala, hypothalamus and cortex [52]; vagal injections and retrograde transport in the locus coeruleus, dorsal raphe nucleus, hypothalamus and amygdala [81]; OB injections and anterograde and retrograde transport in olfactory structures [64] and beyond, including the substantia nigra [63]; and OB/AON injections and retrograde and anterograde transport in olfactory and hippocampal structures [51].

The OB has been identified as a key structure for the prion-like spreading of α-synuclein, as hyposmia is an early manifestation of the disease and Lewy pathology occurs in the OB and AON during Braak’s stage I [65]. The olfactory mucosa is exposed to the environment and directly connected to the OB, which in turn sends projections to the cortex without thalamic relay [31, 50]. Further, the olfactory system is characterized by massive centrifugal projections in which the AON is reciprocally and bilaterally connected [53]. The OB and AON are not only early and preferentially involved in α-synucleinopathy in humans [76, 77, 79], but also in transgenic mouse models [78, 80].

Despite a number of investigations have injected different kinds of α-synuclein in rodents, few of them include injections on OB/AON [51, 63, 64, 74]. In addition, A53T is a suitable model for α-synucleinopathy investigation in olfactory structures [20, 21, 78, 80]. As far as we know, the present report is the first to include injections restricted to the AON in both WT and TG mice.

The aim of this study was, therefore, to validate α-synucleinopathy induction (seeding and spreading) of exogenously injected α-synuclein in the context of prion-like hypothesis in PD [82]. In this study the AON, OB and Pir have been analyzed bilaterally to evaluate possible anterograde and/or retrograde α-synucleinopathy induction including seeding and spreading mechanisms. This is particularly interesting using a model that endogenously overexpress α-synuclein [49] and comparing it with control mice. The concrete objectives of this study were to analyze behavioral changes, connectional spreading, neuronal and glial (microglia and astroglia) involvement after α-synuclein injections in the right AON of both a transgenic PD A53T model (TG) and wild-type mice (WT). To our knowledge this is the first study to include not only WT, but also TG mouse models overexpressing α-synuclein. This allows comparison on the role of endogenously overexpressed human pathological α-synuclein vs. exogenously injected human physiological α-synuclein on seeding and spreading mechanisms along known projections as well as neuronal and glial involvement.

## Material and methods

### Experimental animals

**Fig. 1:**
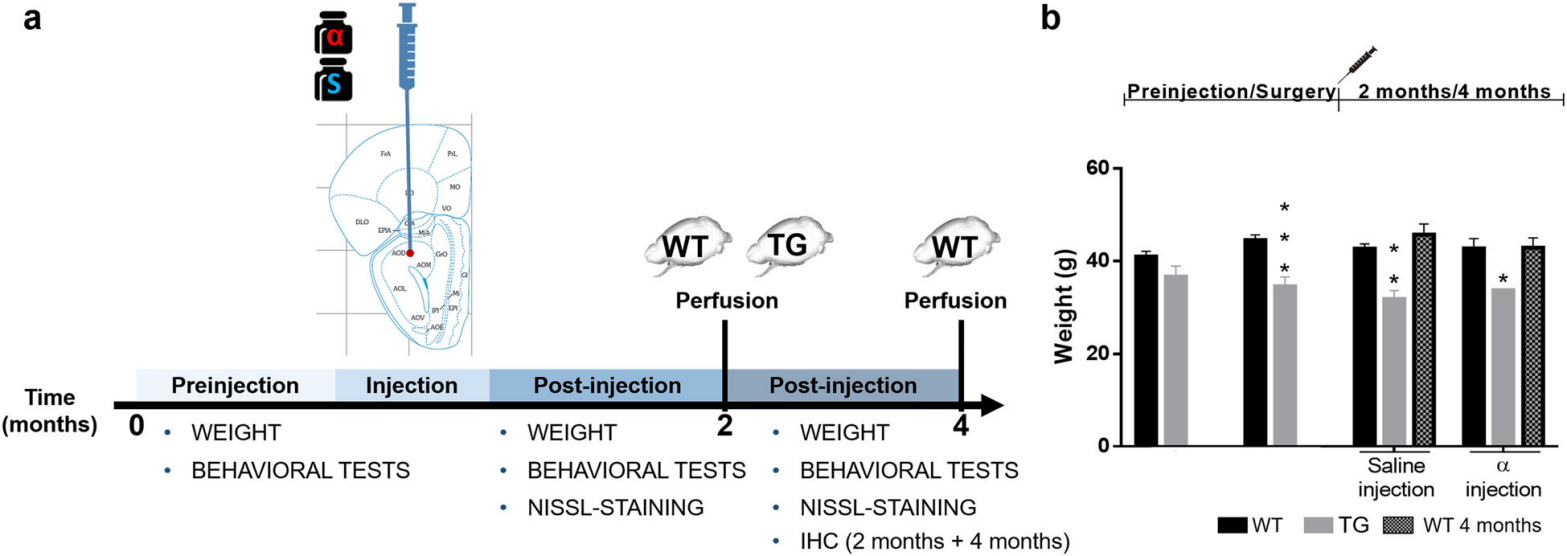
Timeline of experimental design (**a**) and weight measures (**b**). Weight and behavioral measures were taken at different post-injection times: 0, 2 and 4 months. Perfusion of WT and TG mice was carried out after 2 months and another WT group was perfused after 4 months. IHC was performed after 4 months (**a**). Graphs are expressed as mean ± SEM. Two-way ANOVA (Tukey post-hoc tests) showed a decrease in the weight of TG vs. WT. *P < 0.05, **P < 0.01, ***P < 0.0001 (**b**). For abbreviations, see list

Forty-four adult male wild-type (WT, B6C3F1/J) and homozygous transgenic (TG, B6; C3-Tg-Prnp-SNCA*A53T- 83Vle/J; 004479, The Jackson Laboratory, USA) mice were initially used for this study. One group of WT animals was used for tract-tracing experiments. Behavioral analyses were carried out on the remaining animals pre- and post-injection of α-synuclein or saline prior to sacrifice after either 2 or 4 months (Fig. 1a). The initial design of the study thus included nine experimental groups: *n* = 4 for tract-tracing experiments and *n* = 5 each for α-synuclein or saline injections, in TG or WT animals, surviving either 2 or 4 months. At the time of the injection, the average age of animals was 46.63 ± 0.18 (SEM) weeks. Given the high degree of mortality among animals after the injection (especially among TG), TG groups to be sacrificed after 4 months were not included in the final analysis; instead, they were incorporated into the group to be sacrificed after 2 months. Mice were thus divided into seven groups at the end of the investigation. Group 1: WT tract-tracing dextran amine experiments (n = 4); groups 2 and 3: TG α-synuclein (*n* = 3) or saline (n = 4) surviving 2 months; groups 4 and 5: WT α-synuclein (n = 4) or saline (n = 4) surviving 2 months; and groups 6 and 7: WT α-synuclein (n = 4) or saline (n = 5) surviving 4 months; totaling 28 animals at the end of the study. The animals were housed in experimental groups on a standard 12 h/12 h light/dark cycle, at 21 °C, with food and water ad libitum. All experiments carried out were in agreement with European (Directive 2010/63/EU) and Spanish (RD 53/2013) regulations on the protection of animals used for scientific purposes, and they were also approved by the Ethical Committee for Animal Research of the University of Castilla-La Mancha (SAF2016–75768-R).

### Behavioral analyses

#### Corner test

Following the protocol established by [3], a corner test was performed for 30 s. Briefly, animals were individually placed in the center of a clean standard home cage (30.5 cm × 26.5 cm), filled with wood shavings. The number of corners visited, the latency to reach the first corner, the latency to the first rearing and the number of rearings were all recorded and measured.

#### Open-field test

Mice were placed in the center of an open field (a handmade, white wooden box, 55 cm × 55 cm × 25 cm) and observed for 5 min. The following sequence of behavioral events was recorded: latency to leave the central square, latency to enter the peripheral zone, number of rearings and number of crossings. During the test, the numbers of defecation boli and urination episodes were also recorded (data not shown) [3].

#### Rotarod

The LE-8500 rotarod (PanLab/Harvard Apparatus, Barcelona, Spain) was used to examine motor impairment. Each mouse was placed with its head against the direction of rotation. The drum was slowly accelerated from a speed of 4–40 rpm over a 300-s period. The latency to fall from the rotarod within this period was recorded by SeDaCom 2.0 software (PanLab/Harvard Apparatus, Barcelona, Spain). Mice underwent five consecutive trials in one day. The mean latency to fall from the rotarod and the speed for independent and combined trials were analyzed [55, 58].

#### Wire hang test

In the wire hang test, a horizontal wire (diameter 2 mm, length 40 cm) divided into 4 segments was suspended at a height of 80 cm over a cage filled with bedding to avoid injury when falling. The animal was placed clinging to the middle of the wire with its forepaws during one 60-s trial. Motor coordination was measured as the number of segments crossed and muscle strength until falling from the wire [25].

### Tracer injections

Four animals were anesthetized with a combined dose of ketamine hydrochloride (Ketolar, Parke-Davis, Madrid, Spain; 1.5 mL/kg, 75 mg/kg) and xylazine (Xilagesic, Calier, Barcelona, Spain; 0.5 mL/kg, 10 mg/kg). Eye drops (Lacryvisc, Alcon, Barcelona) were applied to prevent eye ulceration during surgery. Under stereotaxic control (anterior 2.8 mm; lateral 1 mm; depth − 2.7 mm, from dura mater) [22], rhodamine-labeled dextran amine (RDA) and fluorescein-labeled dextran amine (FDA) (10,000 mW, lysine fixable, Molecular Probes, Eugene, OR; 10% diluted in PBS) were iontophoretically injected (30–80 μm tip diameter; positive current pulses 7/7 s; 2–7 μA; 8–20 min) at the right and left dorsal AON, respectively.

### α-Synuclein and saline injections

For microinjections, mice were grouped as described above and injected with recombinant α-synuclein (Sigma Aldrich® S7820, human origin, physiological form, N-terminal histidine tagged 140-amino acid protein, 19–20 kDa apparent molecular weight, encoded by a simple gene consisting of six exons on human chromosome 4, expressed in *E. coli,* supplied as lyophilized powder) or saline in the right AON. Animals were anesthetized as described above. Using the same stereotaxic coordinates as used for tracers, 2 μL of 2 μg/μL of α-synuclein in saline or only saline were injected in a constant infusion (0.2 μg per minute) for 10 min using a microsyringe (10 μL Neuros Model 1701 RN, point style 4; SYR, Hamilton Co., Reno, Nevada, USA). Animals were kept on the stereotaxic apparatus for 5 additional minutes to improve the diffusion of the extract before removing the syringe.

### Perfusion and tissue section

Animals injected with tracer were perfused one week post-injection. Animals injected with α-synuclein or saline were perfused either 2 or 4 months after injection. Mice were anesthetized (as described above) and perfused with saline solution followed by 4% *w*/*v* para-formaldehyde fixative (phosphate buffered; 0.1 M sodium phosphate, pH 7.2). Brains were post-fixed in 4% w/v para-formaldehyde and cryoprotected in 30% w/v sucrose. Finally, brains were frontally sectioned using a freezing microtome, and 50-μm sections were sequentially collected in eight-section series.

### Mounting and analyzing sections with fluorescent tracers

Sections were counterstained with 4′6-diamidino-2-phenylindole (DAPI) (Sigma-Aldrich Co. Ltd.) for 5 min in the dark, mounted and coverslipped with antifading polyvinyl alcohol mounting medium (Sigma-Aldrich Co. Ltd.). Images were taken using a confocal microscope LSM 800 (Zeiss, Jena, Germany). Contrast and brightness were adjusted using Image J software.

### Immunohistochemical procedures

For α-synuclein and ionized calcium-binding adaptor molecule 1 (Iba-1) immunohistochemistry, endogenous peroxidase activity was inhibited by bathing twice for 15 min in 1% H_2_O_2_ in phosphate-buffered saline (PBS) (0.01 M pH 7.4). After washes, sections were incubated for 2 h in blocking buffer (0.3% TX-100, 5% normal horse serum with PBS 0.01, pH 7.4). Subsequently, sections were incubated at 4 °C overnight in blocking buffer with primary antibodies (Additional file 1: Table S1). For neuronal marker (NeuN) and glial fibrillary acidic protein (GFAP) immunohistochemistry, endogenous peroxidase activity was inhibited by a 20-min bath in 1% H_2_O_2_ in PBS. After washes, sections were incubated for 2 h in PBS with 0.1% TX-100 (Iba-1) or 0.3% TX-100 (NeuN). Then sections were incubated in PBS with 0.1% TX-100 (Iba-1) or 0.3% TX-100 (NeuN) with primary antibodies at 4 °C overnight (Additional file 1: Table S1).

After washes, sections were incubated for 2 h in secondary biotinylated antibodies in blocking buffer (Additional file 1: Table S1); for NeuN and GFAP, sections were incubated without normal horse serum. Sections for immunohistochemistry were incubated in avidin-biotin complex (ABC Standard, Vector Laboratories), reacted using 0.025% 3,3′-diaminobenzidine and 0.1% H_2_O_2_. Then sections were cresyl violet counterstained (Sigma Aldrich, C5042). The protocol was as follows: dip in distilled H_2_O for 2 min; incubate slides in cresyl violet 0.1% for 1 min; take out the slides, removing the excess by tapping on the container; quickly dip the slides 8 times in 96% ethanol, and then 8 times in 100% ethanol; leave slides in xylene I for 1 min and xylene II for 2 min; coverslip with DPX (Sigma Aldrich, 06522). Controls included omitting primary antibodies, secondary antibodies, and using tissue from WT animals.

### Unbiased, design-based stereology

Quantification was carried out using Stereo Investigator software (MBF Bioscience; microscope Zeiss Axio Imager M2), which is composed of an optical fractionator stereology probe. Stereological techniques are unbiased three-dimensional counting methods using random frames over regions of interest. This probe estimates the number of human α-synuclein aggregates found in cell bodies (aggregates in neurites were not considered), NeuN or Iba-1-positive cells and sub-volumes in the optical dissector (virtual space) in a thick tissue section. Afterwards, these sub-volumes were extrapolated to estimate the entire cell population (cells/mm^3^). The methodology is based on detection of the top-edge of the cell in the dissector and having enough focal planes to determine whether it is in the dissector, using a 63× oil objective and z-axis. This rule is useful to prevent overestimation. Prior to counting aggregates or cells, data such as the number of sections, thickness (50 μm) and interval between sections (eight) were added to the software [26].

OB, AON and piriform cortex (Pir) boundaries were traced using cresyl violet staining at 5× magnification. In addition, OB layers (glomerular, GL; external plexiform, EPL; mitral, MiL; internal plexiform, IPL and granule, GrL layers) were also drawn for Iba-1 staining. Tissue was then examined at high magnification (Plan Apochromat, 63×/ 1.4, Oil lens, Ref: 420782–9900). The parameters of grid size and counting frame were adapted to α-synuclein, NeuN and Iba-1 markers and the size of different layers (Additional file 1: Table S2). Section thickness was measured at each counting site. Both α-synuclein aggregates, NeuN and Iba-1-positive cells were counted by placing a marker on each within the counting frame whilst not touching the red exclusion line.

### Area fraction

Given the number of fibers in GFAP-positive cells, optical dissector analysis was ruled out. The images, taken with a Nikon Eclipse 80i, were captured from each section and hemisphere (OB *n* = 2, 6–9 sections per hemisphere; AON n = 2, 3–4 sections per hemisphere; Pir n = 2, 4–5 sections per hemisphere; 24 animals; *N* = 1550 pictures). Images were processed with ImageJ and converted to 8-bit gray scale. Afterwards, we analyzed the generated histogram. The histogram mode was multiplied by 0.7 to obtain the threshold and measure the area fraction.

### Statistical analysis

Statistical analysis was performed using Graphpad Prism software (v6.01; La Jolla, CA). A Shapiro-Wilk test (*N* < 50) was carried out to analyze the normality. A statistical comparison was analyzed using two-way ANOVA (Tukey post-hoc test) or Kruskal-Wallis non-parametric test (Dunn’s multiple comparisons test), with the following variables: treatment (saline or α-synuclein injection), genotype (WT or TG), time (only for WT, 2 or 4 months survival) or hemisphere (left, LH, or right, RH). Two-tailed and Mann-Whitney tests were only applied when the *P*-value of the source of variation (treatment, genotype, time or hemisphere effect) was significant. Differences were regarded as statistically significant at * or #*P* < 0.05; **or ##*P* < 0.01; *** or ###*P* < 0.001; **** or ####*P* < 0.0001. Significant results are described in the text and/or figures. Statistical data are included in the Additional file 2: Figure S1-S4, Additional file 3: Tables S3–S7 and Additional file 4: Tables S8-S17.

## Results

As described above, although initially nine experimental groups were established, at the end of the investigation only seven were used: one group for tract-tracing experiments and the remaining six groups for α-synuclein or saline injections with different survival times (Fig. 1a). Since behavioral analysis concluded that most effects were due to genotype, but not to experimental procedures, all these results were included in Additional file 2. Similarly, since volume analysis did not report a clear trend, most of these data were included in Additional file 3: Tables S3-S7 and Figure S5. Finally, the injection sites for saline or α-synuclein showed a different appearance depending on the marker used (α-synuclein, Iba-1 or GFAP) (Additional file 4: Figure S6).

### Weight

Animals were weighed during the experimental procedure. Weight loss was observed in TG as compared to WT mice, even before injections (Interaction: F (3, 64) = 1.120, *P* = 0.3478; Treatment effect: F (3, 64) = 0.5025, *P* = 0.6819; Genotype effect: F (1, 64) = 39.07, P < 0.0001). (Fig. 1b). However, no changes were noticed in WT mice as a function of post-injection time (Interaction: F (3, 86) = 0.4156, *P* = 0.7420; Treatment effect: F (3, 86) = 1.975, *P* = 0.1237; Time effect: F (1, 86) = 0.4801, *P* = 0.4902) (Fig. 1b).

### Tract-tracing experiments

**Fig. 2:**
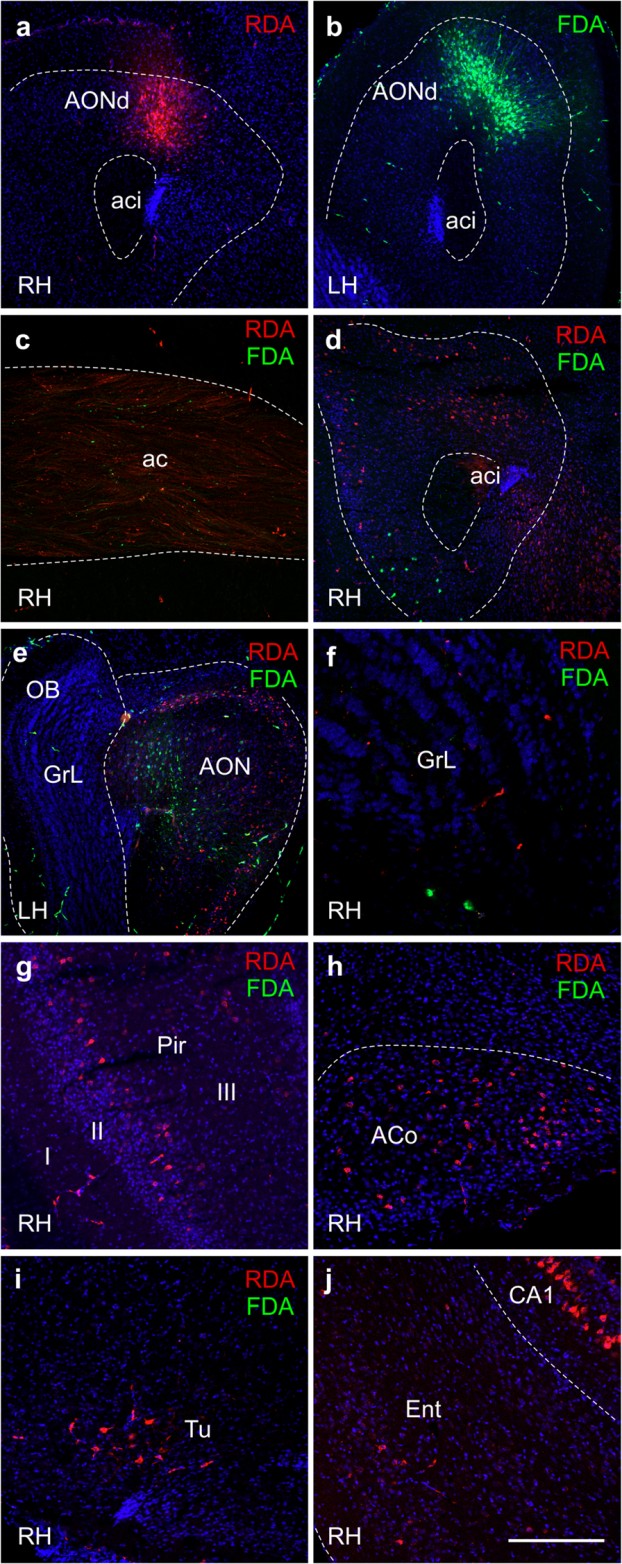
RDA and FDA tracers and DAPI counterstaining. Stereotaxic coordinates were anterior 2.8; lateral 1 and depth − 2.7, from dura mater. **a** RDA injection site in AONd in the right hemisphere. **b** FDA injection site in AONd in the left hemisphere. **c** Interhemispheric fibers were marked by FDA and RDA in the anterior commissure. **d** Example of ipsilateral RDA labeling and contralateral FDA labeling in right AON. **e** Example of ipsilateral FDA labeling and contralateral RDA labeling in left AON. **f** Example of ipsilateral RDA labeling and contralateral FDA labeling in right OB. Most of contralateral labeling from AONd was restricted to ventral regions of AON and closer regions of the granule cell layer (GrL) of the olfactory bulb. Images **g**, **h**, **i** and **j** show usual retrograde ipsilateral labeling in Pir, ACo, Tu and Ent (including pyramidal cells of CA1), respectively. Scale bar: **a**, **b**, **d** and **e** = 400 μm, **c**, **g**-**j** = 200 μm and **f** = 100 μm. For abbreviations, see list

In order to have our own material available to analyze the AON connections, RDA and FDA were injected in the right (Fig. 2a) and left (Fig. 2b) dorsal parts of the AON, respectively. This way, ipsi- and contralateral projections could be easily compared. Axons decussate through the anterior commissure (Fig. 2c). The AON is a secondary olfactory structure and connects to the contralateral AON (Fig. 2d, e). It also connects bilaterally with primary olfactory structures, such as the OB (Fig. 2f) and, more frequently, ipsilaterally with other secondary olfactory structures such as the Pir (Fig. 2g), the anterior cortical amygdala (Fig. 2h), the olfactory tubercle (Fig. 2i) and the entorhinal cortex and hippocampus (Fig. 2j).

### Immunohistochemical analysis

#### α-Synuclein

**Fig. 3:**
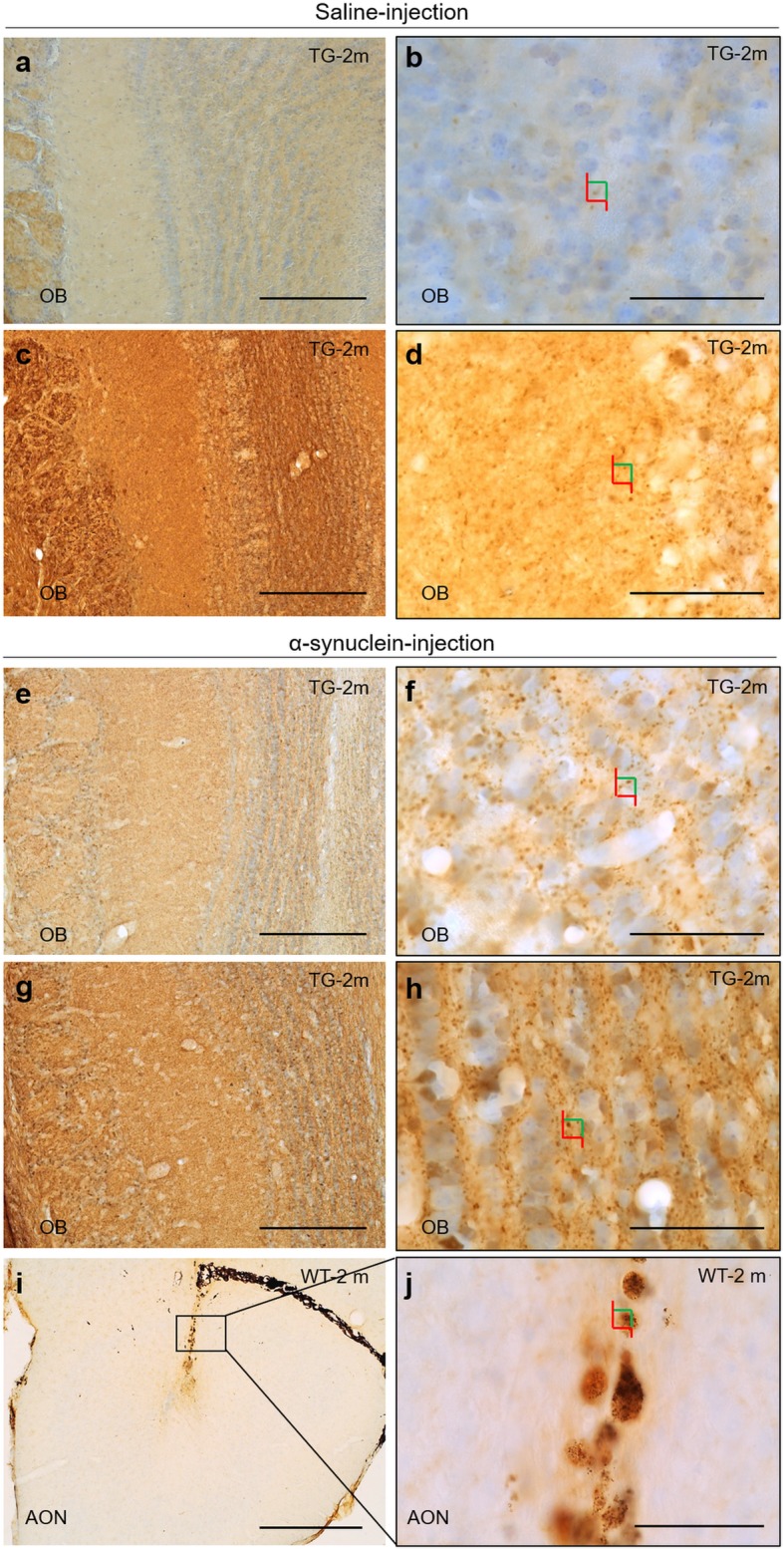
Immunohistochemistry of α-synuclein aggregates in coronal sections of OB (**a**-**h**) and AON (**i**, **j**). Images **b**, **d**, **f**, **h** and **j** are high-magnification of images **a**, **c**, **e**, **g** and **i**, respectively. Colors squares indicate how stereological quantification of aggregates has been performed using optical dissector as shown in Fig. 4. Focusing on saline-injected TG, α-synuclein aggregates were decreased in RH (**a**, **b**) as compared to LH (**c**, **d**). In comparison, α-synuclein injection gave rise higher number of aggregates in RH (**e**, **f**) and particularly in LH (**g**, **h**). In WT, α-synuclein injection was only observed in AON (**i**, **j**). Scale bar **a**, **c**, **e**, **g**: 250 μm; **b**, **d**, **f**, **h**-**j**: 50 μm. For abbreviations, see list

**Fig. 4:**
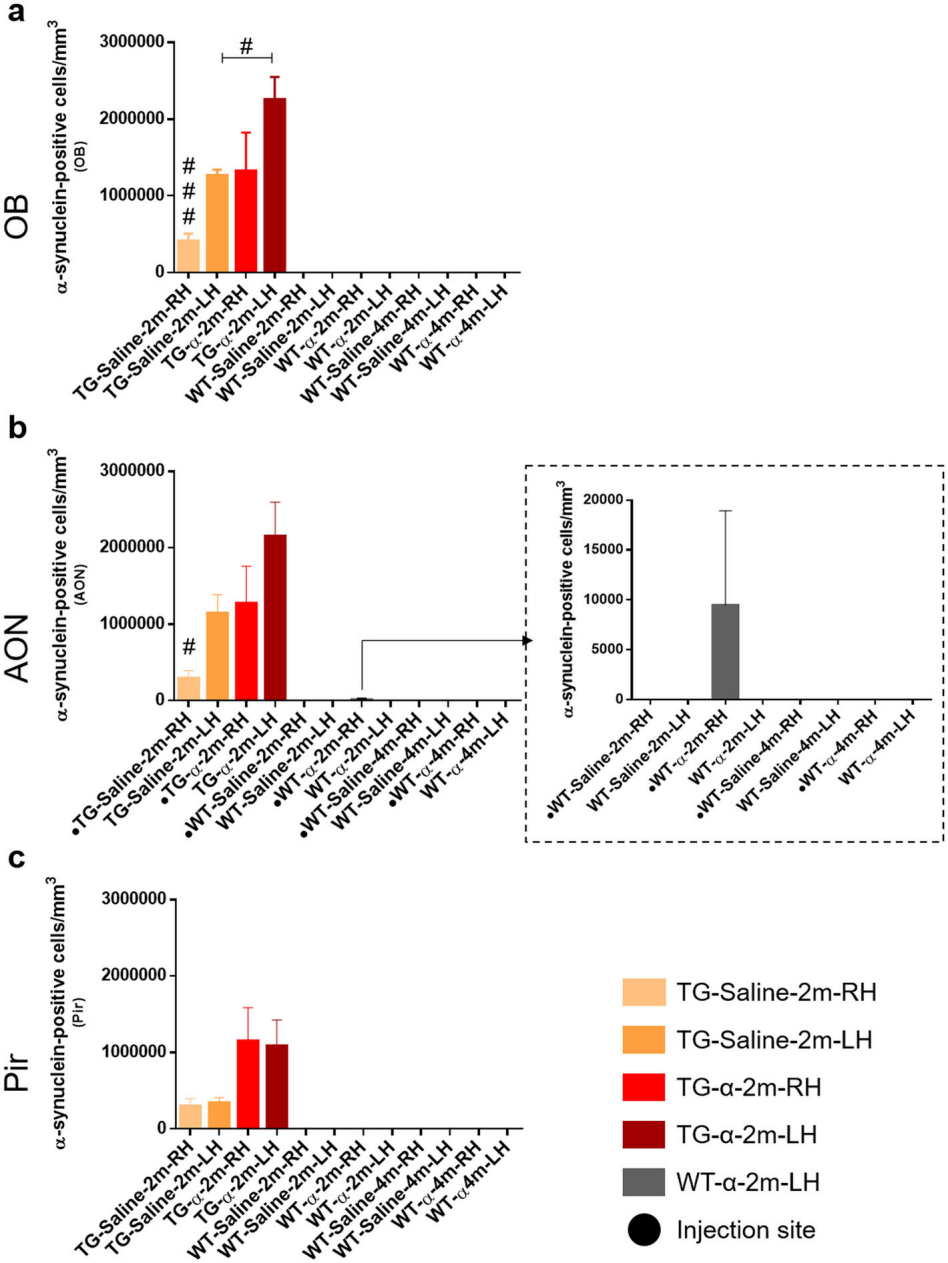
Unbiased stereological quantification of α-synuclein. Graphs (mean ± SEM) represent α-synuclein-positive cells/mm^3^ in OB (**a**), AON (**b**) and Pir (**c**). In graph **b**, arrow indicates counted aggregates in WT, which were only observed in the α-synuclein injection site (dashed square). T-test differences were regarded as statistically significant at #P < 0.05 and ###*P* < 0.001. For statistical data, see Additional file 4: Table S8. For abbreviations, see list

Immunohistochemistry against human α-synuclein revealed both exogenously injected as well as that endogenously overexpressed in TG mice. Endogenous or exogenous α-synuclein cannot be distinguished immunohistochemically. In TG animals, basal α-synuclein expression was observed after saline injection being lower in the RH (Fig. 3a, b) as compared to the LH (Fig. 3 c, d). This difference was statistically significant (Fig. 4a, b). Also, in TG animals, α-synuclein injection in the RH provoked lower α-synuclein expression in the right OB (Fig. 3e, f) as compared to the left OB (Fig. 3g, h). In WT animals only the injection site is labeled (Fig. 3i, j, and dashed square in Fig. 4b).

Stereological analysis revealed variability in the α-synuclein distribution among TG groups. In the OB, lower density was observed in saline-injected mice in the RH as opposed to the LH (t_6_ = 6.948, *P* = 0.0004) (Fig. 4a). Interestingly, in α-synuclein-injected mice higher density was observed in the LH as compared to ipsilaterally in saline-injected mice (t_5_ = 3.805, *P* = 0.0126) (Fig. 4a). In the AON, this trend was maintained but it was only significant between hemispheres in saline-injected animals (TG-Saline-LH vs. TG-Saline-RH: t_6_ = 3.197, *P* = 0.0187; TG-α-LH vs. TG-Saline-LH t_5_ = 2.104, *P* = 0.0893; TG-α-RH vs. TG-Saline-RH t_5_ = 2.310, *P* = 0.0689) (Fig. 4b). This trend was similar but not significant in the Pir (TG-Saline-RH vs. TG-α-syn-RH: t_5_ = 2.225, *P* = 0.0767; TG-Saline-LH vs. TG-α-syn-LH: t_5_ = 2.514, *P* = 0.0536) (Fig. 4c) (Additional file 4: Table S8 and Figure S6).

Even though nigro-striatal projection was not the main goal of this study, histological observations were carried out to check α-synuclein direct or indirect spreading from AON to basal ganglia (caudate-putamen and substantia nigra) (Additional file 4: Figure S7 and S8). α-synuclein aggregates were mostly observed around striosomes (organization of afferent and efferent fibers in the striatum) in the caudate-putamen and substantia nigra compact part in TG animals. In addition, α-synuclein-staining appears to be more intense in α-synuclein injected TG group as compared to saline-injected TG group (Additional file 4: Figure S7 and S8). No differences between hemispheres were observed. These aggregates were not found in WT animals (Additional file 4: Figure S7 and S8).

#### NeuN

Neurodegeneration was evaluated by NeuN staining in OB, AON and Pir. In these areas and bilaterally, NeuN cell density (cells/mm^3^) was significantly higher in saline-injected WT as compared to saline-injected TG group at 2 months (OB: Kruskal-Wallis statistic 22.88, *P* = 0.0018; AON: Kruskal-Wallis statistic 20.27, *P* = 0.0050; Pir: Kruskal-Wallis statistic 23.25, *P* = 0.0015) (Table 1 and Additional file 4: Table S9). No differences between α-synuclein injected WT and TG mice were observed. In right Pir, NeuN cell density significantly decreased in α-synuclein injected WT as compared to saline-injected WT group. This trend, although no significant, was also observed in OB and AON (Table 1 and Additional file 4: Table S9).

**Table 1:** Unbiased stereological quantification of NeuN. Mean ± SEM represent NeuN-positive cells/mm^3^ in the OB, AON and Pir. Statistical analysis was carried out by Kruskal-Wallis and Mann-Whitney tests. For statistical data, see Additional file 4: Table S9. For abbreviations, see list

Brain region	Genotype	Right hemisphere		Left hemisphere	
		Treatment		Treatment	
		Saline-injection	α-synuclein-injection	Saline-injection	α-synuclein-injection
OB	WT	28,894 ± 2616	31,212 ± 1786	34,075 ± 2754	26,836 ± 928.6
	TG	14,729 ± 7364	25,604 ± 1137	17,811 ± 2418	24,450 ± 698.7
AON	WT	19,728 ± 1870	21,405 ± 1990	22,652 ± 2891	21,483 ± 1725
	TG	11,197 ± 1111	17,537 ± 2632	11,680 ± 1329	16,185 ± 722.8
Pir	WT	17,451 ± 501.7	12,642 ± 1001	14,131 ± 13.44	13,375 ± 754.3
	TG	7753 ± 155.8	12,044 ± 778.7	8739 ± 526.3	11,996 ± 588.7

#### Iba-1

**Fig. 5:**
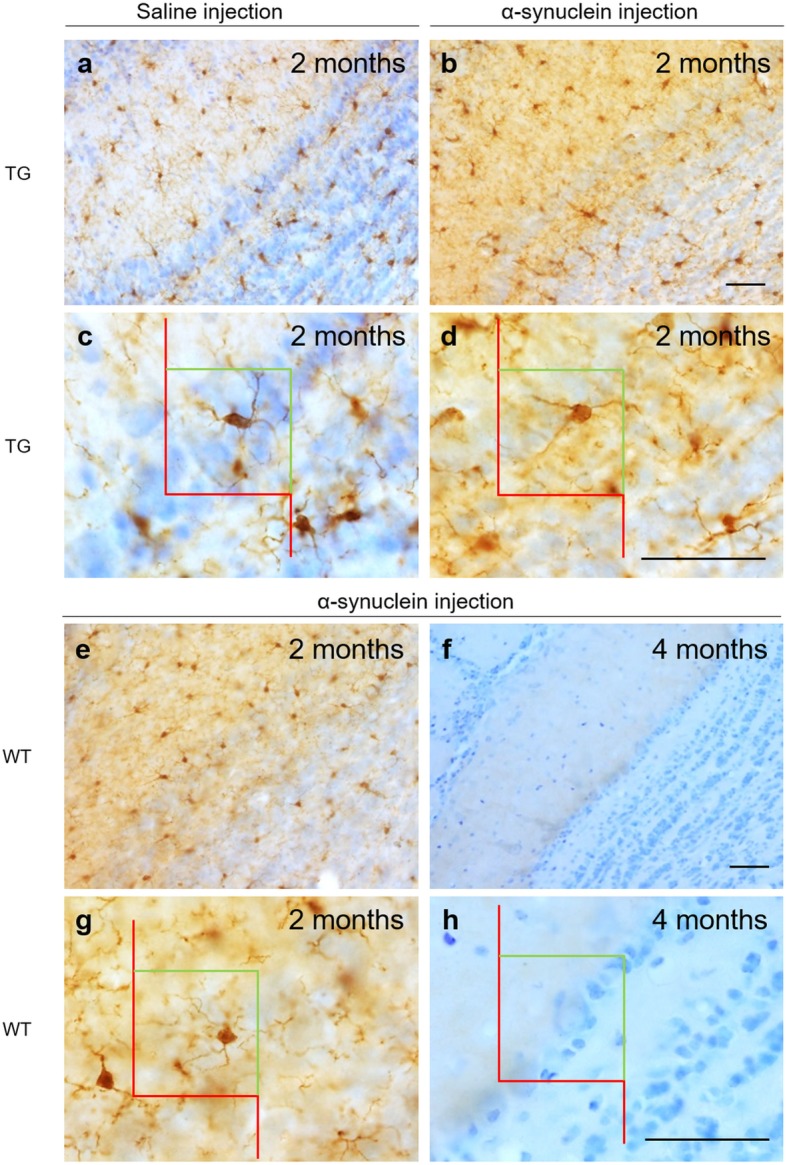
Immunohistochemistry of Iba-1 against coronal sections of saline-injected TG OB (**a**, **c**) and α-synuclein-injected TG OB (**b**, **d**). Different densities between OB WT at 2 months (**e**, **g**) and 4 months (**f**, **h**). Scale bar A–H: 50 μm. For abbreviations, see list

Immunohistochemistry against Iba-1 showed increased labeling in the OB, specifically in the MiL in saline-injected (Fig. 5a, c) vs. α-synuclein-injected (Fig. 5b, d) TG groups. In WT animals, an abrupt reduction of labeling occurred at 4 months (Fig. 5f, h) as compared to 2 months post-injection (Fig. 5e, g). Stereological analysis was applied to the different layers of the OB (GL, EPL, MiL, IPL, GrL), AON and Pir and it was included in Table 2 and Additional file 4: Tables S10-S13. Focusing on RH, Iba-1 density showed no differences in the different layers of OB, AON or Pir, except in MiL. In MiL, saline-injected TG mice had increased Iba-1 density as compared to α-synuclein-injected TG (t_5_ = 3.410; *P* = 0.0190). Also, α-synuclein-injected WT mice showed higher density than α-synuclein-injected TG mice (Interaction: F (1, 11) = 4.943, *P* = 0.0481; Treatment effect: F (1, 11) = 0.8695, *P* = 0.3711; Genotype effect: F (1, 11) = 4.519, *P* = 0.0570). This trend was similar in the LH, revealing a reduction in α-synuclein-injected TG mice as compared to saline-injected TG in MiL (t_5_ = 3.626; *P* = 0.0151) and Pir (t_5_ = 2.620; *P* = 0.0471). In the WT group, post-injection time provoked an abrupt reduction of Iba-1 labeling. There are no differences between hemispheres, whereas lower levels of Iba-1 density were observed in all injected animals (saline or α-synuclein) at 4 months (GL: Interaction: F (3, 26) = 1.253, *P* = 0.3108; Treatment × Time effect: F (3, 26) = 52.42, *P* < 0.0001; Hemisphere effect: F (1, 26) = 0.01173, *P* = 0.9146. EPL: Interaction: F (3, 26) = 1.470, *P* = 0.2456; Treatment × Time effect: F (3, 26) = 82.29, P < 0.0001; Hemisphere effect: F (1, 26) = 0.2745, *P* = 0.6047. MiL. Interaction: F (3, 26) = 0.1273, *P* = 0.9431; Treatment × Time effect: F (3, 26) = 39.19, P < 0.0001; Hemisphere effect: F (1, 26) = 0.1012, *P* = 0.7530. IPL. Interaction: F (3, 26) = 0.1904, *P* = 0.9020; Treatment × Time effect: F (3, 26) = 36.83, P < 0.0001; Hemisphere effect: F (1, 26) = 0.05854, *P* = 0.8107. GrL. Interaction: F (3, 26) = 1.304, *P* = 0.2944; Treatment × Time effect: F (3, 26) = 149.7, P < 0.0001; Hemisphere effect: F (1, 26) = 1.179, *P* = 0.2876. AON. Interaction: F (3, 26) = 1.304, P = 0.2944; Treatment × Time effect: F (3, 26) = 149.7, P < 0.0001; Hemisphere effect: F (1, 26) = 1.179, P = 0.2876. Pir. Interaction: F (3, 26) = 0.06495, *P* = 0.9779; Treatment × Time effect: F (3, 26) = 35.75, P < 0.0001; Hemisphere effect: F (1, 26) = 0.001407, *P* = 0.9704). Moreover, all injected animals showed no differences between hemispheres in the TG group as well as in WT mice (Table 2, Additional file 4: Tables S10–S13 and Figure S6).

**Table 2:** Unbiased stereological quantification of Iba-1. Mean ± SEM represent Iba-1-positive cells/mm^3^ in the OB layers, AON and Pir. Statistical analysis was focused on comparisons: genotype in the right hemisphere, left hemisphere, interhemispheric differences in WT and TG. For statistical data, see Additional file 4: Tables S10-S13. For abbreviations, see list

Iba-1 positive cells (cells/mm^3^)						
Brain region	Genotype	Post-injection time	Right hemisphere		Left hemisphere	
			Treatment		Treatment	
			Saline-injection	α-synuclein-injection	Saline-injection	α-synuclein-injection
GL	WT	2 m	8600 ± 475	9726 ± 1253	10,463 ± 931	8519 ± 1543
		4 m	1359 ± 199.1	3028 ± 618.1	856 ± 348.1	2617 ± 613.9
	TG	2 m	7583 ± 778.3	9249 ± 2388	7860 ± 548	10,569 ± 1451
EPL	WT	2 m	8260 ± 1297	9412 ± 538.3	8828 ± 594.3	7514 ± 1019
		4 m	833.4 ± 124.5	1086 ± 234.2	639.5 ± 303.8	1613 ± 582.3
	TG	2 m	7421 ± 782	8102 ± 482	7543 ± 531	6762 ± 544
MiL	WT	2 m	8939 ± 2174	10,807 ± 1040	8921 ± 1831	9984 ± 1285
		4 m	935.6 ± 449.1	1162 ± 289.6	314.3 ± 98.62	1599 ± 642.4
	TG	2 m	9080 ± 521	4513 ± 1421	10,296 ± 549	6338 ± 1048
IPL	WT	2 m	12,870 ± 3512	11,089 ± 1404	13,703 ± 279.3	10,294 ± 1572
		4 m	1134 ± 402.8	1066 ± 306.5	948.7 ± 481.7	2209 ± 1070
	TG	2 m	14,126 ± 1671	8969 ± 4074	12,480 ± 2987	9101 ± 3479
GrL	WT	2 m	9897 ± 361.6	10,597 ± 733.6	10,044 ± 321.80	8839 ± 1185
		4 m	827.8 ± 230.5	1301 ± 364.3	265.6 ± 62.71	1659 ± 662.9
	TG	2 m	8646 ± 876	11,231 ± 1751	9316 ± 934	10,632 ± 567
AON	WT	2 m	10,536 ± 1506	10,687 ± 2337	10,031 ± 1196	8168 ± 1690
		4 m	700.3 ± 425.8	555.7 ± 385.8	0 ± 0	321.1 ± 230.7
	TG	2 m	8665 ± 1015	8084 ± 2493	8810 ± 703	9251 ± 1681
Pir	WT	2 m	8202 ± 1371	8798 ± 1763	8395 ± 471.5	8241 ± 2339
		4 m	108 ± 74.58	330.7 ± 143.7	0 ± 0	684.8 ± 569.9
	TG	2 m	7151 ± 882	9312 ± 1061	9025 ± 546	6999 ± 507

#### GFAP

**Fig. 6:**
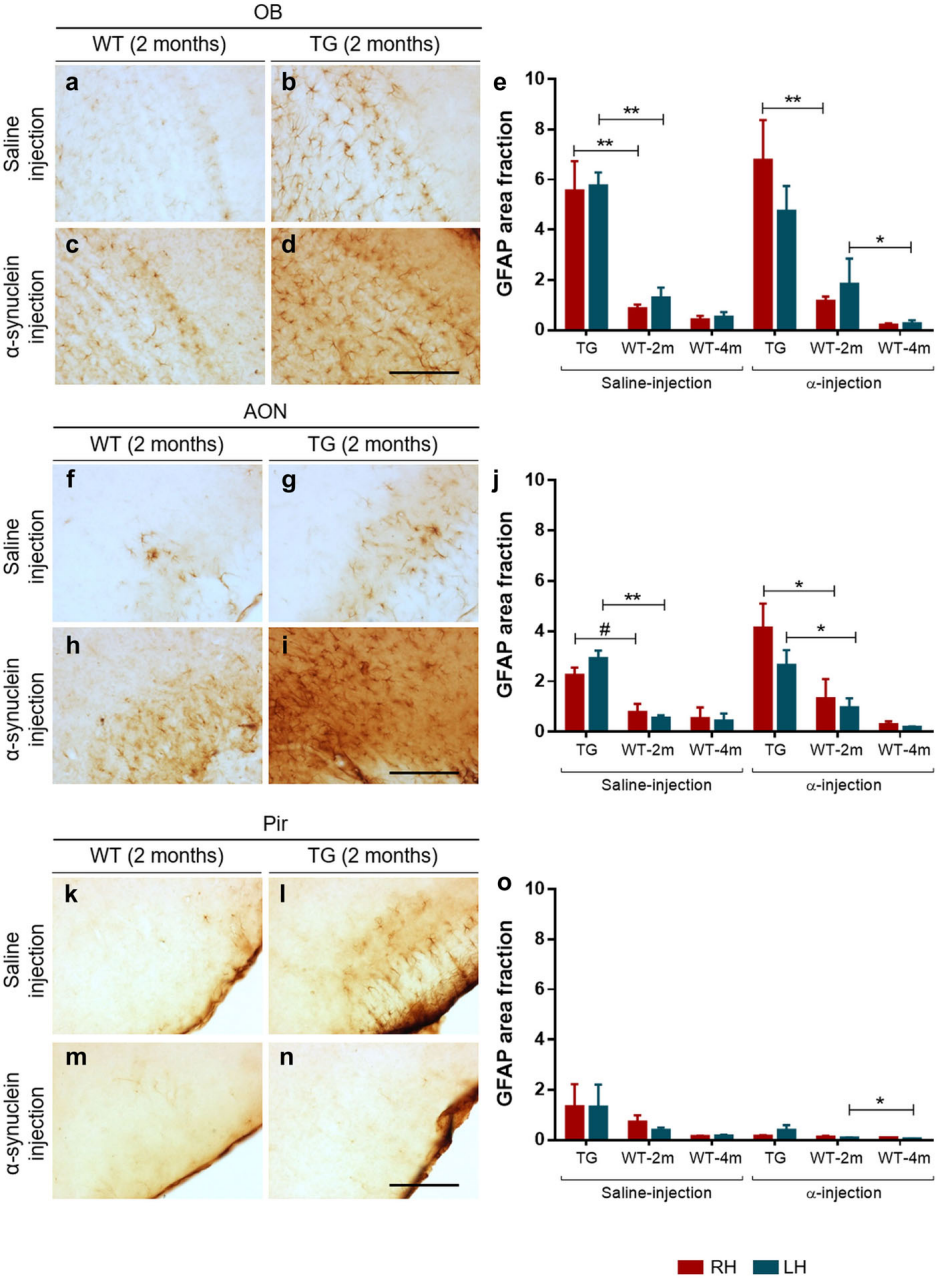
Immunohistochemistry of GFAP (**a**–**d**, **f**–**i**, **k**–**n**) and area fraction data (**e**, **j**, **o**) in coronal sections of OB, AON and Pir. Statistical analysis was focused on comparisons: genotype in the right hemisphere, left hemisphere, interhemispheric differences in WT and TG. For statistical data, see Additional file 4: Tables S14-S17. Scale bar: 125 μm. For abbreviations, see list

GFAP immunohistochemistry revealed astroglial morphology in the different layers of the OB (Fig. 6a–d), AON (Fig. 6f–i) and Pir (Fig. 6k–n) in the different experimental groups. The complex morphology of GFAP-positive cells has led us to use area fraction instead of stereological analysis for quantification. Two-way ANOVA and two-tailed t-test showed significantly higher GFAP area fraction in all injected TG (saline and α-synuclein) as compared to all WT in the right OB (Fig. 6e) (Interaction: F (1, 11) = 0.2590, *P* = 0.6208; Treatment effect: F (1, 11) = 0.6954, *P* = 0.4221; Genotype effect: F (1, 11) = 32.13, *P* = 0.0001), and AON (Fig. 6j) (Interaction: F (1, 11) = 1.121, *P* = 0.3123; Treatment effect: F (1, 11) = 3.740, *P* = 0.0793; Genotype effect: F (1, 11) = 11.73, *P* = 0.0057. t_6_ = 3.134; *P* = 0.0202). However, the genotype effect was maintained in AON (Fig. 6j) (Interaction: F (1, 11) = 0.8663, *P* = 0.3720; Treatment effect: F (1, 11) = 0.03401, *P* = 0.8570; Genotype effect: F (1, 11) = 30.14, *P* = 0.0002) and OB (Fig. 6e) (Interaction: F (1, 11) = 0.9921, *P* = 0.3406; Treatment effect: F (1, 11) = 0.08698, *P* = 0.7735; Genotype effect: F (1, 11) = 22.10, *P* = 0.0006) except in α-synuclein-injected TG mice in OB in the LH. No differences were observed in Pir (Fig. 6o). Focusing on WT groups, α-synuclein-injected animals showed a significant GFAP area fraction reduction in left OB (Fig. 6e) (Interaction: F (3, 26) = 0.2841, *P* = 0.8364; Treatment effect: F (3, 26) = 4.233, *P* = 0.0146; Hemisphere effect: F (1, 26) = 1.315, *P* = 0.2619) and Pir (Fig. 6o) (Interaction: F (3, 26) = 1.279, *P* = 0.3024; Treatment effect: F (3, 26) = 2.273, *P* = 0.1038; Hemisphere effect: F (1, 26) = 0.6240, *P* = 0.4367) at 4 months but not in AON (Fig. 6j). Finally, there were no differences in TG groups when comparing hemispheres (Fig. 6e, j, o) (Additional file 4: Tables S14–S17 and Figure S6).

## Discussion

This study analyzes behavioral changes, connectional seeding, spreading, neuronal and glial involvement after α-synuclein injections in the right AON of TG and WT mice. Behavioral analysis reveals, in general, higher levels of hyperactivity and better motor coordination in TG as compared to WT mice (Additional file 2). Brain volume analysis detects multiple changes across the experimental groups, but no clear trend could be extrapolated (Additional file 3: Tables S3-S7). Tract-tracing experiments show that the main afferent contralateral projections to the AONd come from the AON and secondarily from the OB (Fig. 2). Regarding synucleinopathy in TG mice, two main conclusions were reached: first, in saline-injected animals, α-synuclein expression in OB and AON is higher in LH as compared to RH, which can be attributable to basal interhemispheric differences; second, in the OB, α-synuclein injection appears to induce a significant increase in the LH as compared to saline-injected animals, which can be due to retrograde, contralateral, hodological α-synucleinopathy induction (Figs. 3 and 4). Neurodegeneration, estimated by the number of NeuN-positive cells/mm^3^, is observed in saline-injected TG animals as compared to saline-injected WT group at 2 months. In Pir, NeuN-positive cells/mm^3^ decrease in α-injected WT vs. saline-injected WT group (Table 1 and Additional file 4: Table S9). Changes observed in microglia can be attributed to the effect of the injection (Fig. 5, Table 2 and Additional file 4: Tables S10–S13). Finally, astroglia increase in TG mice independently of experimental conditions likely provoked by endogenous α-synucleinopathy, whereas in WT the α-synuclein injection provokes astrogliosis that is reverted at 4 months post-injection (Fig. 6 and Additional file 4: Tables S14–S17). Therefore, this study suggests α-synucleinopathy induction along known pathways, particularly via retrograde transport and through contralateral projections, within the olfactory system of transgenic animals.

### Connections of the anterior olfactory nucleus

The AON is preferentially involved in proteinopathies at an early stage, not only in PD [2, 15, 72, 76, 80], but also in Alzheimer’s disease [68], and it has been hypothesized that this is due to its multiple connections [67, 79]. The human AON is a structure composed of several subdivisions [77], whose connections are only inferred in the human brain from comparative data [79], which is scarce in primates [54] but more abundant in rodents [9, 53]. The results presented here (Fig. 2) confirm that the AON is a secondary bulbar structure, reciprocally and bilaterally connected to other secondary olfactory structures, and also to at least 27 non-olfactory structures [9]. Ipsilaterally, the AON is reciprocally connected to the OB [53] (Fig. 2). Contralaterally, afferent projections to the AONd are mainly originated in the AON and secondarily in the OB (Fig. 2). Accordingly, contralateral and trans-synaptic involvement or related connections should be considered in our α-synucleinopathy results. Spreading from AON to basal ganglia, including substantia nigra and caudate-putamen, cannot discarded via indirect pathways [79].

### α-Synuclein

In order to evaluate α-synuclein spreading along the olfactory anatomical pathways [79], α-synuclein or saline were injected into the right AON. In fact, this nucleus was chosen because it could be the starting point for spreading the pathology in the brain. Actually, although analyzing nigro-striatal projection involvement was not the main objective of the study, exciting findings were reached (Additional file 4: Figure S7 and S8). Our observations confirmed that α-synucleinopathy was present in caudate-putamen and, in contrast to the original description of A53T mouse model, also in the substantia nigra [24]. Interestingly, this α-synucleinopathy in A53T model appears to be enhanced by α-synuclein injection. These aggregates were not found in WT animals (Additional file 4: Figure S7 and S8).

Although multiple studies have injected different kinds of α-synuclein in mice (preformed fibrils, oligomers, human extracts and sick mice extracts), those focused on OB/AON injections are scarce [51, 63, 64, 74]. On the other hand, A53T is a useful α-synucleinopathy model that expresses α-synuclein in olfactory structures. Previous reports of our work describe maximum synucleinopathy levels over 40 weeks [20, 21, 78, 80]. As far as we know, the present report is the first to include injections restricted to the AON in both WT and TG mice. Our results show that α-synuclein aggregates were mostly found in the contralateral hemisphere (left OB and AON) after saline injections, which could be attributable to basal interhemispheric differences (Fig. 4). This observation is very relevant in the context of unresolved motor asymmetry reported in PD [16]. Another interesting observation is that there is a trend for a denser labeling in the contralateral hemisphere of α-synuclein-injected TG as compared to saline-injected TG animals, particularly in the OB (Fig. 4). This could be related to the previously described contralateral projections (Fig. 2), indicating α-synucleinopathy retrograde induction (seeding and/or spreading). Endogenous α-synuclein expressed in TG animals would be necessary to allow exogenous injected α-synuclein to increase synucleinopathy in the contralateral hemisphere. Thus, we suggest that exogenous α-synuclein is able to induce seeding and/or spreading to the contralateral hemisphere in TG animals. In WT animals, α-synuclein was only detected at the injection site. These results could be due to different factors: susceptibility to a prior seeding, time-dependent spreading or reactivity of different molecular α-synuclein species. First, it has been reported that α-synuclein preformed fibrils-inoculated A53T mice enhanced the conversion of endogenous α-synuclein into pathological forms, acting as a template. This has been reported not only in vivo, but also in vitro, since α-synuclein aggregation could occur as a nucleation-dependent mechanism [48]. Second, the post-injection periods vary in different studies, ranging from 72 h to 30, 90 or 180 days [48, 64, 66]. Third, despite α-synuclein oligomerizing and aggregating into fibrils under pathological conditions, it has been reported that monomers and oligomers were more rapidly transferred from OB of WT to interconnected regions as compared to fibrils [64]. Therefore, it would be interesting to have this kind of molecule available to improve the injection results; α-synuclein recombinants are larger, which possibly makes spreading more difficult, thus requiring more time. In agreement with our results, α-synuclein fibrils have been intrahippocampally injected in TG and WT mice, without α-synuclein spread from the injection site having been detected in WT after 2 months [66].

### NeuN

PD is characterized by neurodegeneration of dopaminergic neurons in the substantia nigra pars compacta and the corresponding denervation in the striatum [39]. Interestingly, in patients, this has been correlated with neuronal loss in the OB [35].

Neurodegeneration has been checked using NeuN labeling in different mouse PD models, including 6-OHDA injections [46], adeno-associated-virus vector (AAV9) for α-synuclein [57] and transgenic mouse overexpressing human α-synuclein under the Thy-1 promoter (Thy-α-syn) [11]. Bilaterally, in OB, AON and Pir, our results showed that the number of NeuN-positive cells/mm^3^ (density) was higher in saline-injected WT group as compared to saline-injected TG mice at 2 months. Given that these differences are not observed in α-synuclein injected animals, we suggested that, in WT mice, α-synuclein injection might be also involved in neurodegeneration. In fact, in Pir, NeuN density decreased in α-synuclein injected WT as compared to saline-injected WT group.

No differences were noticed in TG animals, regardless of injection and hemispheres. It could be due to the fact that A53T mouse model constitutively expresses human α-synuclein gene, therefore α-synuclein injection could provoke minor induction on neurodegeneration. As far as we know, this is the first report describing unbiased NeuN counting in olfactory structures in A53T mouse model.

Regarding α-synucleinopathy and neurodegeneration, despite of our results show basal interhemispheric differences in α-synuclein aggregates in saline-injected TG in OB and AON, there is not correlation to neuronal loss. Likewise, although data show an increase of α-synuclein between saline-injected TG and α-synuclein-injected TG in left OB, no differences were observed in NeuN counting.

The density of NeuN differences between saline-injection TG and saline-injection WT could be due to transgene. These differences disappeared in α-synuclein injected animals. So, it could be as a consequence of exogenous α-synuclein inoculation. In WT mice, although our findings indicated that α-synuclein was restricted to the injection site (right AON), neuronal loss was significantly observed in Pir. It could be due to the fact that our α-synuclein antibody is able to label α-synuclein aggregates but no other conformations such as fibrils or soluble forms, which could act on neurodegeneration as well.

### Glial markers

Both microglia and astroglia have been involved in PD progression [32] and associated α-synucleinopathy [7].

#### Microglia

Microglia are actively involved both in healthy brains and in different neurodegenerative disorders such as Parkinson’s and Alzheimer’s diseases [69]. Microglia play multiple roles in neuronal survival, not only in physiological inflammatory responses but also in non-inflammatory ones. For example, their involvement in homeostasis, programmed cell death and clearance of apoptotic newborn neurons has been reported, as well as synaptic pruning and synaptic plasticity [69]. Focusing on PD, neuroinflammation has been described not only as a consequence of neurodegeneration, but also as an issue at the onset of this disease [45]. As previous studies have shown that the brains of PD patients have extensive microglial activation [45], one of the aims of this study was to quantify microglia (Iba-1-positive cells) in an A53T mouse model and WT mice to detect microgliosis after saline or α-synuclein injections. Surprisingly, our results only showed a reduction of Iba-1-positive cells in α-synuclein-injected TG mice as compared to saline-injected TG or α-synuclein-injected WT in the MiL and Pir (Fig. 5 and Table 2). This might be due to the fact that TG mice have endogenous human α-synuclein and, as we injected with a similar molecular species, it was not detected as exogenous. On the other hand, our results have shown a significant time-dependent decrease after 4 months in WT (Fig. 5 and Table 2). Thereby, the injection/surgery might have triggered a small amount of microgliosis. The effect of microgliosis in PD remains controversial because it can be both beneficial and detrimental [45]. In addition, we assessed differences in microglial morphology (larger cell bodies and shorter and thicker dendrites) that were correlated to the detection of homeostatic changes (image not shown).

#### Astroglia

Astrocytes are the largest cells in the brain. Their critical functions include maintaining a homeostatic microenvironment (modulating oxidative stress and regulating glucose metabolism), neuronal survival, neurotransmission and injury repair, among others [32, 37]. Interestingly, it has been proposed that astrocytes can establish communications with neurons, microglia and other astrocytes. In fact, it has been suggested that one of the likely causes of neuronal death could be astrocyte dysfunction [37] and also that activated astrocytes can prevent neuronal death. Indeed, published reports indicate that PD genes are expressed in both astrocytes and microglia, and that astrogliosis is activated in PD. Astroglia protect neurons from oxidative stress and inhibit excessive inflammation by regulating microglial activation. Taking all this into consideration, one of the aims of this study was to evaluate the GFAP area fraction in all injected animals. Our results indicated that the GFAP area fraction increased in all injected TG (saline or α-synuclein) as compared to all WT in the right OB and AON (Fig. 6e, j). In agreement with these results, non-injected TG mice showed a higher number of GFAP-positive cells in the striatum and substantia nigra as compared to WT [73]. However, the GFAP area fraction decreased at 4 months in WT mice (Fig. 6e, j, o). Therefore, injection/surgery could have triggered astrogliosis as well as microgliosis in WT mice.

## Conclusions

Being aware of limitations of study due to lethality, the main conclusions reached are the followings. In TG saline-injected animals, α-synuclein expression in OB and AON is higher in LH as compared to RH (Fig. 4 a and b); and, in the OB, α-synuclein injection could provoke a significant increase in the LH as compared to saline-injected animals (Fig. 4a). This study suggests connectional seeding and/or spreading of α-synuclein along known olfactory pathways of TG animals, particularly via retrograde transport and through AON to the contralateral OB (Fig. 2). Neurodegeneration might be correlated with transgene and exogenous α-synuclein inoculation could induce neuronal loss in WT animals, even though this α-synucleinopathy cannot be detected (Table 1 and Additional file 4). Microglia labeling was apparently correlated with surgery-induced inflammation (Fig. 5, Table 2 and Additional file 4). On the other hand, astroglial labeling was higher in TG animals, which could be due to endogenous α-synucleinopathy; whereas in WT the α- synuclein injection could provoke astrogliosis that is reverted at 4 months post-injection (Fig. 6 and Additional file 4). Behavioral changes observed might be mostly attributable to genotype (Additional file 2).

## Additional files

Additional file 1: Material and methods. **Table S1.** Antibodies. **Table S2.** Parameters of unbiased, design-based stereology. (PDF 123 kb)

Additional file 2: Behavioral analyses. **Figure S1.** Corner test. **Figure S2.** Open field test. **Figure S3** Rotarod test. **Figure S4.** Wire hang test. (PDF 948 kb)

Additional file 3: Brain volume. **Table S3.** Brain volume. **Table S4.** Volume statistical data. Comparison of genotype in the right hemisphere. **Table S5.** Volume statistical data. Comparison of genotype in the left hemisphere. **Table S6.** Volume statistical data. Comparison of hemispheres in WT. **Table S7.** Volume statistical data. Comparison of hemispheres in TG. **Figure S5.** OB and AON volume 3D model. (PDF 233 kb)

Additional file 4: Stereological α-synuclein, NeuN, Iba-1 and GFAP quantification. **Table S8.** Statistical data of stereological α-synuclein quantification. **Table S9.** Statistical data of NeuN quantification (Mann-Whitney test). **Table S10.** Statistical data of stereological Iba-1 quantification. Comparison of genotype in the right hemisphere. **Table S11.** Statistical data of stereological Iba-1 quantification. Comparison of genotype in the left hemisphere. **Table S12.** Statistical data of stereological Iba-1 quantification. Comparison of hemispheres in WT. **Table S13.** Statistical data of stereological Iba-1 quantification. Comparison of hemispheres in TG. **Table S14.** Statistical data of GFAP quantification. Comparison of genotype in the right hemisphere. **Table S15.** Statistical data of GFAP quantification. Comparison of genotype in the left hemisphere. **Table S16.** Statistical data of GFAP quantification. Comparison of hemispheres in WT. **Table S17.** Statistical data of GFAP quantification. Comparison of hemispheres in TG. **Figure S6.** AON injection site labeled with different markers. **Figure S7.** α-synucleinopathy in caudate-putamen. **Figure S8.** α-synucleinopathy in substantia nigra. (PDF 814 kb)

## Abbreviations

ABC: Avidin-biotin complex
ac: Anterior commissure
aci: Anterior commissure intrabulbar
ACo: Anterior cortical amygdala
AOD: Anterior olfactory area dorsal part
AOL: Anterior olfactory area lateral part
AOM: Anterior olfactory area medial part
AON: Anterior olfactory nucleus
AONd: Dorsal anterior olfactory nucleus
AOV: Anterior olfactory area ventral part
CA1: Field CA1 of Ammon’s horn
Ent: Entorhinal cortex
EPL: External plexiform layer
FDA: Fluorescein-labeled dextran amine
g: Grams
GFAP: Glial fibrillary acidic protein
GL: Glomerular layer
GrL: Granule cell layer
Iba-1: Ionized calcium binding adaptor molecule 1
IH: Interhemispheric
IHC: Immunohistochemistry
IPL: Internal plexiform layer
LH: Left hemisphere
m: Months
MiL: Mitral cell layer
mm: Millimeter
OB: Olfactory bulb
PBS: Phosphate-buffered saline
PD: Parkinson’s disease
Pir: Piriform cortex
RDA: Rhodamine-labeled dextran amine
RH: Right hemisphere
rpm: Revolutions per minute
S: Saline solution
s: Seconds
SNC: Substantia nigra, compact part
SNR: Substantia nigra, reticular part
TG: Transgenic
Tu: Olfactory tubercle
WT: Wild-type
α: α-synuclein
α-syn: α-synuclein
