# Supplementary material for: Neurodegeneration and contralateral α-synuclein induction after intracerebral α-synuclein injections in the anterior olfactory nucleus of a Parkinson’s disease A53T mouse model
Source: Acta Neuropathol Commun. 2019 Apr 15;7:56. doi: 10.1186/s40478-019-0713-7 (PMC6463651; doi:10.1186/s40478-019-0713-7)
Supplement: Supplementary file 1 — Material and methods. Table S1. Antibodies. Table S2. Parameters of unbiased, design-based stereology. (PDF 123 kb) [file 40478_2019_713_MOESM1_ESM.pdf]

ADDITIONAL FILE 1

Neurodegeneration and contralateral α-synuclein induction after intracerebral α-synuclein injections in the anterior olfactory nucleus of a Parkinson's disease A53T mouse model

Alicia Flores-Cuadrado[1], Daniel Saiz-Sanchez[1], Alicia Mohedano-Moriano[2], Alino Martinez-Marcos[1], Isabel Ubeda-Bañon[1]*

[1]Neuroplasticity and Neurodegeneration Laboratory, CRIB, Ciudad Real Medical School, University of Castilla-La Mancha, Ciudad Real, Spain.

[2]School of Occupational Therapy, Speech Therapy and Nursing, University of Castilla-La Mancha, Talavera de la Reina, Spain.

Alicia.flores@uclm.es
Daniel.saiz@uclm.es
Alicia.mohedano@uclm.es
Alino.martinez@uclm.es

Address for correspondence:
Isabel Ubeda-Bañon
University of Castilla-La Mancha
Ciudad Real Medical School
Camino de Moledores s/n
13071 Ciudad Real (Spain)
Phone: 926295300 6835
E-mail Isabel.ubeda@uclm.es

Material and methods

Table S1. Antibodies

Antigen	Manufacturer, cat nº, species	Dilution	Secondary antibodies
α-synuclein (KM51)	Novocastra™(Leica Biosystems), NCL-L-ASYN, mouse	1:50	1:200 biotinylated horse anti-mouse IgG (H+L) Vector laboratories
Iba-1	Wako, 019-19741, rabbit	1:500	1:200 biotinylated horse anti-rabbit IgG (H+L) Vector laboratories
GFAP	BD Pharmingen™, 556329, mouse	1:5000	1:200 biotinylated horse anti-mouse IgG (H+L) Vector laboratories
NeuN	Abcam ab104225, rabbit	1:1000	1:200 biotinylated horse anti-rabbit IgG (H+L) Vector laboratories

Table S2. Parameters of unbiased, design-based stereology

Area	Counting frame α-synuclein (μm x μm)	Counting frame Iba-1 (μm x μm)	Counting frame NeuN (μm x μm)	Grid size α-synuclein (μm x μm)	Grid size NeuN (μm x μm)	Grid size Iba-1 (μm x μm)
OB	7 x 7	–	20 x 20	200 x 200	233 x 484	–
GL	–	50 × 50	-	–	-	8 × 8
EPL	–	50 × 50	-	–	-	8 × 8
IPL	–	50 × 50	-	–	-	20 × 20
MiL	–	50 × 50	-	–	-	20 × 20
GrL	–	50 × 50	-	–	-	8 × 8
AON	7 x 7	50 × 50	20 x 20	200 × 200	122 x 221	5 × 5
Pir	7 x 7	50 × 50	20 x 20	200 × 200	233 x 484	5 × 5
