# Supplementary material for: Neurodegeneration and contralateral α-synuclein induction after intracerebral α-synuclein injections in the anterior olfactory nucleus of a Parkinson’s disease A53T mouse model
Source: Acta Neuropathol Commun. 2019 Apr 15;7:56. doi: 10.1186/s40478-019-0713-7 (PMC6463651; doi:10.1186/s40478-019-0713-7)
Supplement: Supplementary file 2 — Behavioral analyses. Figure S1. Corner test. Figure S2. Open field test. Figure S3 Rotarod test. Figure S4. Wire hang test. (PDF 948 kb) [file 40478_2019_713_MOESM2_ESM.pdf]

ADDITIONAL FILE 2

Neurodegeneration and contralateral α-synuclein induction after intracerebral α-synuclein injections in the anterior olfactory nucleus of a Parkinson's disease A53T mouse model

Alicia Flores-Cuadrado[1], Daniel Saiz-Sanchez[1], Alicia Mohedano-Moriano[2], Alino Martinez-Marcos[1], Isabel Ubeda-Bañon[1]*

[1]Neuroplasticity and Neurodegeneration Laboratory, CRIB, Ciudad Real Medical School, University of Castilla-La Mancha, Ciudad Real, Spain.

[2]School of Occupational Therapy, Speech Therapy and Nursing, University of Castilla-La Mancha, Talavera de la Reina, Spain.

Alicia.flores@uclm.es
Daniel.saiz@uclm.es
Alicia.mohedano@uclm.es
Alino.martinez@uclm.es

Address for correspondence:
Isabel Ubeda-Bañon
University of Castilla-La Mancha
Ciudad Real Medical School
Camino de Moledores s/n
13071 Ciudad Real (Spain)
Phone: 926295300 6835
E-mail Isabel.ubeda@uclm.es

Behavioral analyses

Supplementary Figures and Figures legends

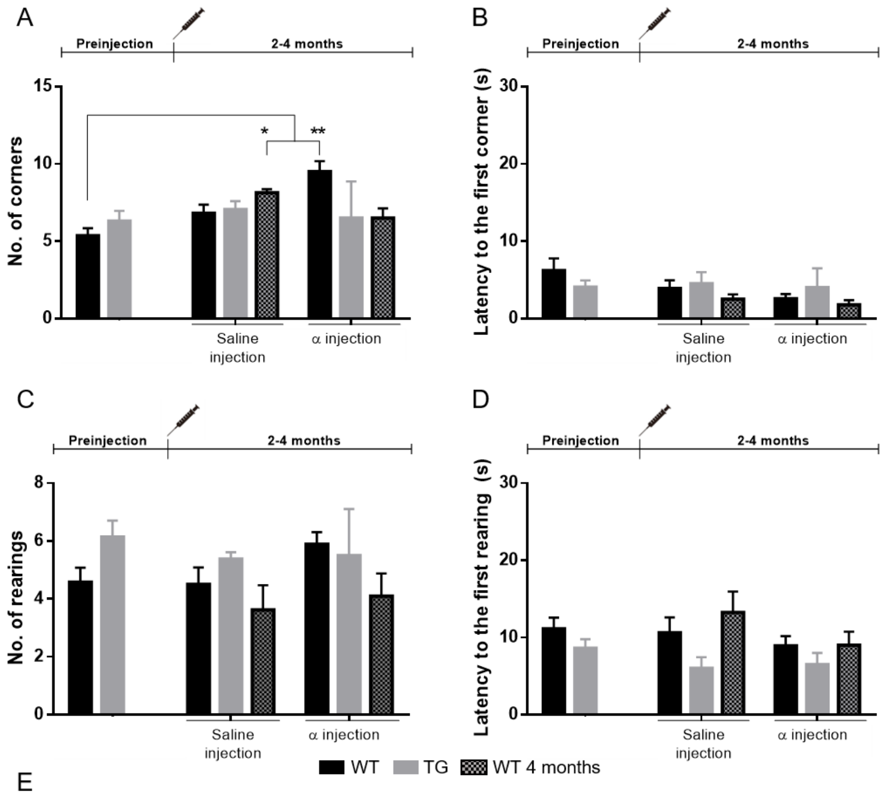

E

Corner test			
Figure	Source of variation	F (DFn, DFd)	P value
Figure 3A	Interaction	F (2, 54) = 2.713	P = 0.0754
	Treatment	F (2, 54) = 2.876	P = 0.0651
	Genotype	F (1, 54) = 0.6704	P = 0.4165
	Interaction	F (2, 51) = 4.512	P = 0.0157*
	Treatment	F (2, 51) = 7.198	P = 0.0018**
	Time	F (1, 51) = 0.8772	P = 0.3534
Figure 3B	Interaction	F (2, 54) = 0.5458	P = 0.5825
	Treatment	F (2, 54) = 0.5278	P = 0.5929
	Genotype	F (1, 54) = 0.0005	P = 0.9826
	Interaction	F (2, 56) = 0.0622	P = 0.9397
	Treatment	F (2, 56) = 2.325	P = 0.1071
	Time	F (1, 56) = 0.1986	P = 0.6575
Figure 3C	Interaction	F (2, 54) = 0.7534	P = 0.4756
	Treatment	F (2, 54) = 0.4453	P = 0.6429
	Genotype	F (1, 54) = 1.075	P = 0.3044
	Interaction	F (2, 56) = 0.8246	P = 0.4437
	Treatment	F (2, 56) = 0.8991	P = 0.4127
	Time	F (1, 56) = 2.438	P = 0.1241
Figure 3D	Interaction	F (2, 54) = 0.1846	P = 0.8320
	Treatment	F (2, 54) = 0.5958	P = 0.5547
	Genotype	F (1, 54) = 3.700	P = 0.0597
	Interaction	F (2, 56) = 0.2668	P = 0.7668
	Treatment	F (2, 56) = 1.123	P = 0.3324
	Time	F (1, 56) = 0.2843	P = 0.5960

Fig. S1 Corner test. Graphics illustrating the entries to corner (A), latency to the first corner (B), rearings (C) and latency to the first rearing (D) for 30 seconds, and statistical data (E). Results are

presented as mean ± SEM. Corner test was used to measure neophobia and the data showed that entries to corners (A) were increased in α-synuclein-injected WT at 2 months (black bar) and saline-injected WT at 4 months (grid bar) as compared to pre-injected WT. No differences were observed from “latency to the first corner” (B), rearings (C) and “latency to the first rearing” (D). For abbreviations, see list.

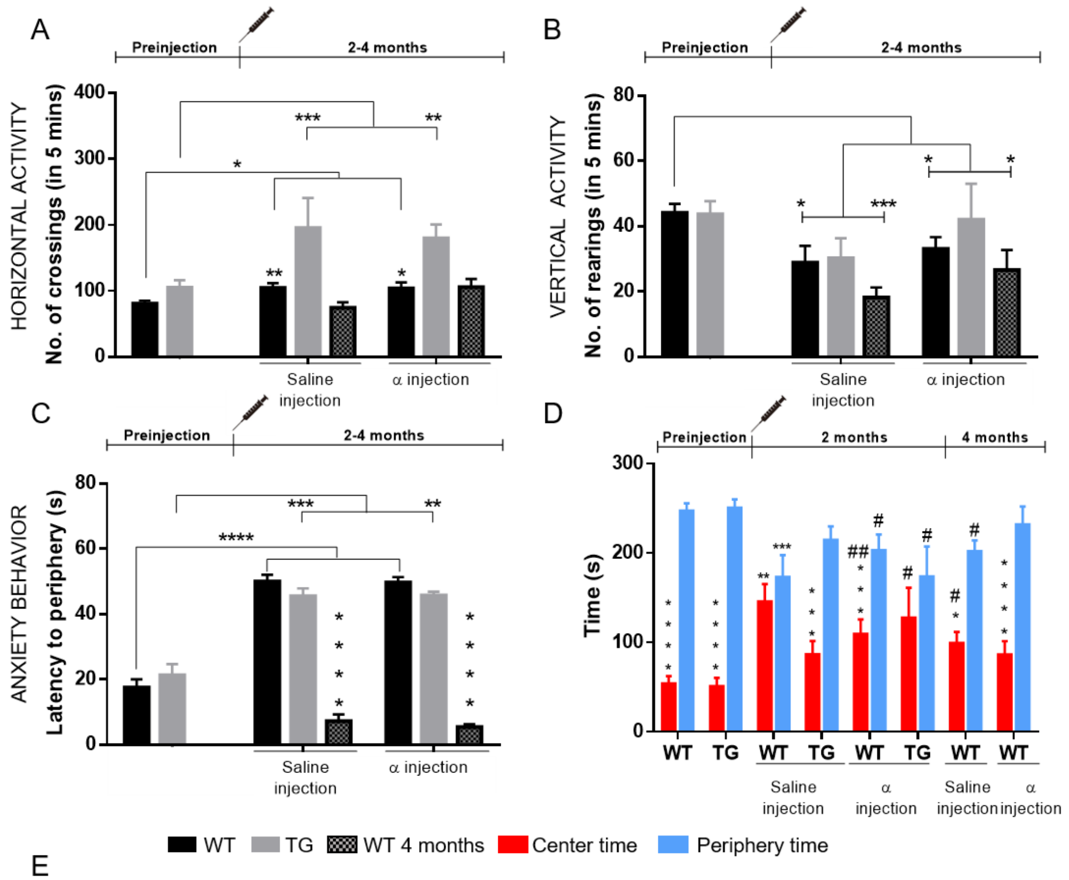

E

Open field test			
**Figure**	**Source of variation**	**F (DFn, DFd)**	**P value**
**Figure 4A**	Interaction	F (2, 54) = 2.556	P = 0.0870
	Treatment	F (2, 54) = 8.041	P = 0.0009***
	Genotype	F (1, 54) = 25.63	P < 0.0001****
	Interaction	F (2, 56) = 2.640	P = 0.0802
	Treatment	F (2, 56) = 4.767	P = 0.0122*
	Time	F (1, 56) = 2.175	P = 0.1458
**Figure 4B**	Interaction	F (2, 54) = 0.3995	P = 0.6726
	Treatment	F (2, 54) = 3.341	P = 0.0429*
	Genotype	F (2, 54) = 0.5098	P = 0.4783
	Interaction	F (2, 56) = 0.7703	P = 0.4677
	Treatment	F (2, 56) = 11.99	P < 0.0001****
	Time	F (1, 56) = 2.626	P = 0.1107
**Figure 4C**	Interaction	F (2, 54) = 0.6377	P = 0.5325
	Treatment	F (2, 54) = 31.59	P < 0.0001****
	Genotype	F (1, 54) = 0.2212	P = 0.6400
	Interaction	F (2, 56) = 30.70	P < 0.0001****
	Treatment	F (2, 56) = 7.231	P = 0.0016**
	Time	F (1, 56) = 122.7	P < 0.0001****
**Figure 4D**	Interaction	F (7, 119) = 5.456	P < 0.0001****
	Genotype x treatment	F (7, 119) = 0.093	P = 0.9986 (t)
	Center time x Periphery time	F (1, 119) = 152.8	P < 0.0001****(t)

(t) Center time: WT pre-WT α: t_{24} = 3.229, P = 0.0036##; WT pre-WT saline 4m: t_{20} = 2.285, P = 0.0334#; TG pre-TGα: t_{19} = 2.704, P = 0.0141#; Periphery time: WT pre-WTα: t_{25} = 2.458, P = 0.0213#; WT pre-WT saline 4m: t_{20} = 2.285, P = 0.0334#; TG pre-TGα: t_{19} = 2.705, P = 0.0140#.

Fig. S2 Open field test. Horizontal activity was measured by the number of crossings (A) and vertical activity was analyzed by the number of rearings (B). Latency to periphery (C) and time in the center and periphery of the open field box (D) were used to assess anxiety-like behavior. Graphs are represented as mean ± SEM. Statistical data are presented as a table (E). All injected mice (saline and α-synuclein) crossed a greater number of squares than pre-injected animals (A). After injection, TG animals (grey bar) crossed a greater number of times as compared to WT animals (black bar) (A). On the other hand, all injected WT mice (saline or α-synuclein) developed lower rearings than pre-injected WT mice (B). This difference was also observed in all injected WT mice at 4 months post-injection (grid bar) (B). All injected animals showed anxiety-like behavior, measured as "latency of periphery," as compared to pre-injected animals (C). All groups spent more time in the periphery zone (D). For abbreviations, see list.

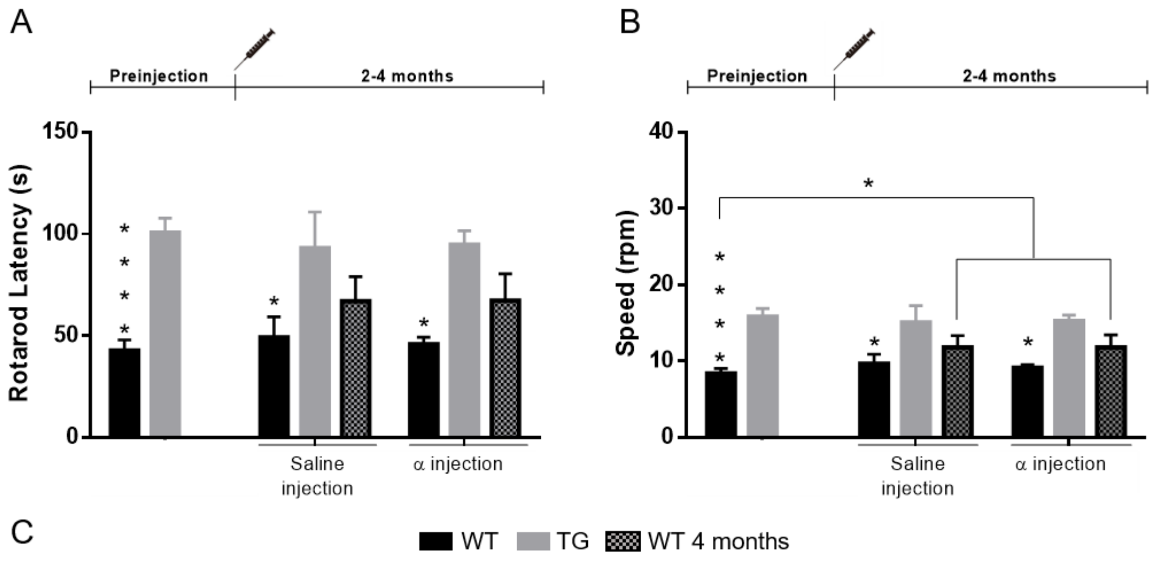

C

Rotarod			
**Figure**	**Source of variation**	**F (DFn, DFd)**	**P value**
**Figure 5A**	Interaction	F (2, 53) = 0.2640	P = 0.7690
	Treatment	F (2, 53) = 0.0095	P = 0.9905
	Genotype	F (1, 53) = 38.83	P < 0.0001****
	Interaction	F (2, 56) = 0.9487	P = 0.3934
	Treatment	F (2, 56) = 2.063	P = 0.1366
	Time	F (1, 56) =3.687	P = 0.0599
**Figure 5B**	Interaction	F (2, 53) = 0.3071	P = 0.7369
	Treatment	F (2, 53) = 0.0190	P = 0.9812
	Genotype	F (1, 53) = 33.94	P < 0.0001****
	Interaction	F (2, 56) = 0.9427	P = 0.3957
	Treatment	F (2, 56) = 3.174	P = 0.0495*
	Time	F (1, 56) = 3.641	P = 0.0615

Fig. S3 Rotarod test. Balance and motor coordination were evaluated by rotarod latency to fall (A) and speed at the time of falling (B) over 300 seconds and 5 consecutive trials. Results are presented as mean ± SEM. For statistical data, see C. The latency to fall of all TG mice (grey bar) were greater than for WT groups (A). In WT groups (black bar), no differences were observed in the latency regarding time (A), though speed was greater in injected animals at 4 months (grid bar) post-injection (B). For abbreviations, see list.

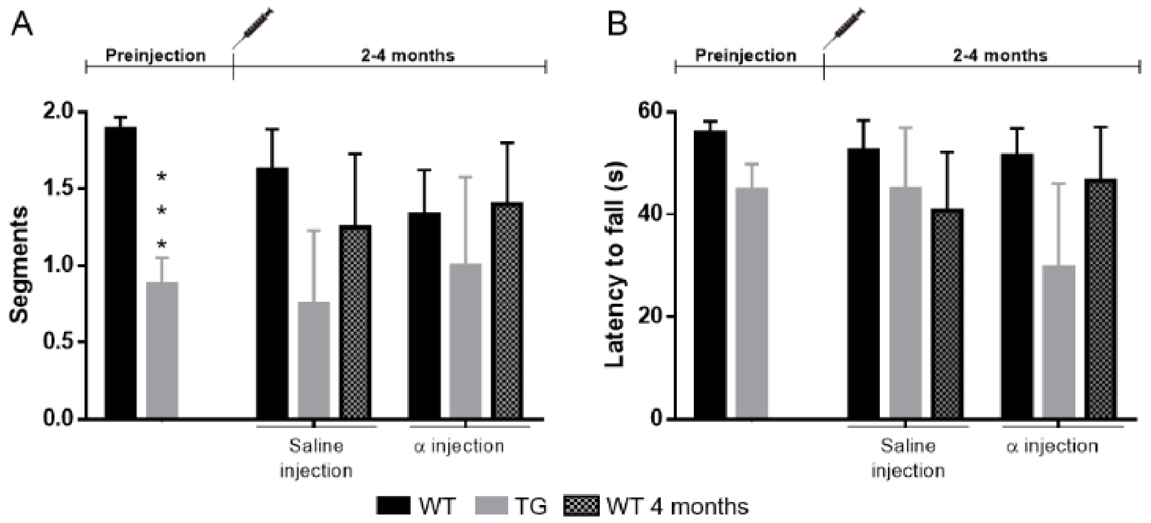

C

Wire hang			
**Figure**	**Source of variation**	**F (DFn, DFd)**	**P value**
**Figure 6A**	Interaction	F (2, 53) = 0.8978	P = 0.4136
	Treatment	F (2, 53) = 0.4121	P = 0.6643
	Genotype	F (1, 53) = 11.51	P = 0.0013**
	Interaction	F (2, 56) = 0.6173	P = 0.5432
	Treatment	F (2, 56) = 3.493	P = 0.0372*
	Time	F (1, 56) = 0.3438	P = 0.5600
**Figure 6B**	Interaction	F (2, 53) = 0.5972	P = 0.5540
	Treatment	F (2, 53) = 1.204	P = 0.3082
	Genotype	F (1, 53) = 5.902	P = 0.0185*
	Interaction	F (2, 56) = 0.6769	P = 0.5123
	Treatment	F (2, 56) = 1.817	P = 0.1720
	Time	F (1, 56) = 1.781	P = 0.1874

Fig. S4 Wire hang test. Motility was measured by segments crossed (A) and strength was analyzed by latency to fall (B) over 60 seconds. Results are presented as mean ± SEM. For statistical data, see C. Pre-injected TG group (grey bar) showed less motility (covered segments) than the WT group (black bar) (A). Non-significant changes were observed post-injection in their motility (A) and strength (B). For abbreviations, see list.

Discussion

Our results reveal pre-injection weight loss in TG mice that remained constant post-injection as a consequence of genotype effect (Fig. 1B) as described previously (Giasson et al., 2002; Paumier et al., 2013; Guerreiro et al., 2017). The first characterization of the A53T mice model determined severe and complex motor impairment, and even paralysis (Giasson et al., 2002). For this study, our tests were focused on motor impairments, anxiety and neophobia. Corner test data showed no differences in vertical activity (rearings), while horizontal activity (visited corners) increased in α-synuclein-injected WT at 2 months and saline-injected WT at 4 months as compared to pre-injected WT (Fig. S1). These behavioral results indicate that animals did not exhibit neophobia, since a reduction in the visited corners or rearings was not observed. A53T mice exhibited hyperactive behavior as they crossed more squares in the open field test than WT mice, independently of the injection used (saline or α-synuclein) (Fig. S2). This is in agreement with previous data on the distance traveled in the open field test (Paumier et al., 2013). However, other authors have indicated that A53T mice showed significantly reduced locomotor activity from 2 to 12 months (Oaks et al., 2013). Evaluation of the open field data suggests that A53T and WT mice spent more time in the periphery, although the latency to periphery is higher in injected animals (Fig. S2C, D). At this point the explanation of these results becomes complex, because it could be that the animals were moving more either because they had become more accustomed to the cage or possibly because of their hyperactivity. Indeed, these results are correlated with increased time spent in the center zone by TG (Fig. S2D) (Oaks et al., 2013).

Despite previous suggestions that balance and coordination (rotarod test) were reduced in both WT and TG mice as a function of age (Oaks et al., 2013; Paumier et al., 2013), there was no indication of this in our study (Fig. S3). Our results were largely consistent with previous reports in which A53T exhibited better results in terms of latency and speed than WT mice (Paumier et al., 2013; Guerreiro et al., 2017), which contrasts with other reports where A53T and WT mice show different scores (Oaks et al., 2013). Strength and motility were indexed by the wire hang test. A53T mice traveled fewer segments and latency to fall tended to decrease (Fig. S4), which is in agreement with other reports (Oaks et al., 2013). Our global data suggest that the behavioral changes observed are due to genotype effect rather than experimental conditions.

References

Giasson BI, Duda JE, Quinn SM, Zhang B, Trojanowski JQ, Lee VM. 2002. Neuronal alpha-synucleinopathy with severe movement disorder in mice expressing A53T human alpha-synuclein. Neuron 34(4):521-533.

Guerreiro PS, Coelho JE, Sousa-Lima I, Macedo P, Lopes LV, Outeiro TF, Pais TF. 2017. Mutant A53T alpha-Synuclein Improves Rotarod Performance Before Motor Deficits and Affects Metabolic Pathways. Neuromolecular Med 19(1):113-121.

Oaks AW, Frankfurt M, Finkelstein DI, Sidhu A. 2013. Age-dependent effects of A53T alpha-synuclein on behavior and dopaminergic function. PLoS One 8(4):e60378.

Paumier KL, Sukoff Rizzo SJ, Berger Z, Chen Y, Gonzales C, Kaftan E, Li L, Lotarski S, Monaghan M, Shen W, Stolyar P, Vasilyev D, Zaleska M, W DH, Dunlop J. 2013. Behavioral characterization of A53T mice reveals early and late stage deficits related to Parkinson's disease. PLoS One 8(8):e70274.
