# Supplementary material for: Neurodegeneration and contralateral α-synuclein induction after intracerebral α-synuclein injections in the anterior olfactory nucleus of a Parkinson’s disease A53T mouse model
Source: Acta Neuropathol Commun. 2019 Apr 15;7:56. doi: 10.1186/s40478-019-0713-7 (PMC6463651; doi:10.1186/s40478-019-0713-7)
Supplement: Supplementary file 3 — Brain volume. Table S3. Brain volume. Table S4. Volume statistical data. Comparison of genotype in the right hemisphere. Table S5. Volume statistical data. Comparison of genotype in the left hemisphere. Table S6. Volume statistical data. Comparison of hemispheres in WT. Table S7. Volume statistical data. Comparison of hemispheres in TG. Figure S5. OB and AON volume 3D model. (PDF 233 kb) [file 40478_2019_713_MOESM3_ESM.pdf]

ADDITIONAL FILE 3

Neurodegeneration and contralateral α-synuclein induction after intracerebral α-synuclein injections in the anterior olfactory nucleus of a Parkinson's disease A53T mouse model

Alicia Flores-Cuadrado[1], Daniel Saiz-Sanchez[1], Alicia Mohedano-Moriano[2], Alino Martinez-Marcos[1], Isabel Ubeda-Bañon[1]*

[1]Neuroplasticity and Neurodegeneration Laboratory, CRIB, Ciudad Real Medical School, University of Castilla-La Mancha, Ciudad Real, Spain.

[2]School of Occupational Therapy, Speech Therapy and Nursing, University of Castilla-La Mancha, Talavera de la Reina, Spain.

Alicia.flores@uclm.es
Daniel.saiz@uclm.es
Alicia.mohedano@uclm.es
Alino.martinez@uclm.es

Address for correspondence:
Isabel Ubeda-Bañon
University of Castilla-La Mancha
Ciudad Real Medical School
Camino de Moledores s/n
13071 Ciudad Real (Spain)
Phone: 926295300 6835
E-mail Isabel.ubeda@uclm.es

Brain volume

Table S3 Brain volume. Estimated volume of OB and layers (GL, EPL, MiL, IPL and GrL), AON and Pir (mm^3). Statistical analysis was focused on comparisons: genotype in the right hemisphere, left hemisphere, interhemispheric differences in WT and TG. For statistical data, see tables S4–S7. For abbreviations, see list.

Brain volume **(mm^3).**						
Brain region	Genotype	Post-injection time	Right hemisphere		Left hemisphere	
			Treatment		Treatment	
			Saline-injection	α-synuclein-injection	Saline-injection	α-synuclein-injection
GL	WT	2m	1.266 ± 0.16240	1.429 ± 0.21380	1.079 ± 0.1874	1.707 ± 0.1439
		4m	1.331 ± 0.03881	1.366 ± 0.08751	1.296 ± 0.1084	1.330 ± 0.0528
	TG	2m	1.531 ± 0.101	1.879 ± 1.030	1.677 ± 0.092	1.649 ± 0.275
EPL	WT	2m	2.036 ± 0.47050	1.461 ± 0.42040	1.507 ± 0.204	1.979 ± 0.075
		4m	1.513 ± 0.02033	1.845 ± 0.27060	1.479 ± 0.108	1.821 ± 0.182
	TG	2m	2.212 ± 0.173	1.741 ± 0.517	2.076 ± 0.097	1.886 ± 0.691
MiL	WT	2m	0.6628 ± 0.3200	0.3823 ± 0.0618	0.5561 ± 0.25680	0.4575 ± 0.02483
		4m	0.5617 ± 0.2111	0.6692 ± 0.3375	0.4080 ± 0.09933	0.5087 ± 0.17860
	TG	2m	0.4404 ± 0.0128	1.0520 ± 0.4581	0.4541 ± 0.0263	0.7220 ± 0.1527
IPL	WT	2m	0.5496 ± 0.26080	0.8661 ± 0.58200	0.2748 ± 0.02186	0.3440 ± 0.02245
		4m	0.2705 ± 0.01046	0.5900 ± 0.30690	0.2503 ± 0.01836	0.5450 ± 0.18600
	TG	2m	0.3313 ± 0.0177	0.7728 ± 0.3827	0.4943 ± 0.2031	1.5640 ± 0.6492
GrL	WT	2m	2.480 ± 0.31330	2.081 ± 0.39640	2.315 ± 0.3042	2.713 ± 0.1151
		4m	2.045 ± 0.04228	2.048 ± 0.14200	1.999 ± 0.1459	2.152 ± 0.1120
	TG	2m	2.082 ± 0.164	1.850 ± 0.539	2.034 ± 0.103	2.078 ± 0.338
OB	WT	2m	6.777 ± 0.7947	5.319 ± 0.9610	5.624 ± 0.8263	7.075 ± 0.5384
		4m	5.476 ± 0.1643	5.867 ± 0.2115	5.603 ± 0.1494	5.375 ± 0.6235
	TG	2m	6.664 ± 0.271	7.619 ± 0.465	6.416 ± 0.166	6.906 ± 0.254
AON	WT	2m	1.4140 ± 0.19500	1.9620 ± 0.30250	1.4110 ± 0.24660	1.4240 ± 0.17510
		4m	1.3340 ± 0.10540	1.1830 ± 0.09017	1.2420 ± 0.07154	1.2480 ± 0.06981
	TG	2m	1.4080 ± 0.0741	0.9065 ± 0.3043	1.358 ± 0.239	1.795 ± 0.391
Pir	WT	2m	3.3170 ± 0.1805	2.9090 ± 0.2370	3.5160 ± 0.1474	3.1360 ± 0.2130
		4m	2.6530 ± 0.1506	3.0530 ± 0.1288	2.9120 ± 0.2310	3.3500 ± 0.1018
	TG	2m	3.7330 ± 0.1305	3.0950 ± 0.6904	3.66400 ± 0.1970	3.52700 ± 0.44470

Table S4. Volume statistical data. Comparison of genotype in the right hemisphere.

Area	Source of variation	F (DFn, DFd)	P value
GL	Interaction	F (1, 11) = 0.04726	P = 0.8319
	Treatment	F (1, 11) = 0.3606	P = 0.5604
	Genotype	F (1, 11) = 0.7059	P = 0.4187
EPL	Interaction	F (1, 11) = 0.01629	P = 0.9007
	Treatment	F (1, 11) = 1.648	P = 0.2256
	Genotype	F (1, 11) = 0.3132	P = 0.5869
MiL	Interaction	F (1, 11) = 3.187	P = 0.1018
	Treatment	F (1, 11) = 0.4390	P = 0.5213
	Genotype	F (1, 11) = 0.8012	P = 0.3899
IPL	Interaction	F (1, 11) = 0.02753	P = 0.8712
	Treatment	F (1, 11) = 1.012	P = 0.3360
	Genotype	F (1, 11) = 0.1711	P = 0.6871
GrL	Interaction	F (1, 11) = 0.05523	P = 0.8185
	Treatment	F (1, 11) = 0.7884	P = 0.3936
	Genotype	F (1, 11) = 0.7834	P = 0.3950
OB	Interaction	F (1, 11) = 2.837	P = 0.1202
	Treatment	F (1, 11) = 0.1233	P = 0.7321
	Genotype	F (1, 11) = 2.331	P = 0.1551
AON	Interaction	F (1, 11) = 5.140	P = 0.0445*
	Treatment	F (1, 11) = 0.01009	P = 0.9218
	Genotype	F (1, 11) = 5.258	P = 0.0425*
Pir	Interaction	F (1, 11) = 0.1301	P = 0.7252
	Treatment	F (1, 11) = 2.690	P = 0.1292
	Genotype	F (1, 11) = 0.8912	P = 0.3654

Table S5. Volume statistical data. Comparison of genotype in the left hemisphere.

Area	Source of variation	F (DFn, DFd)	P value
GL	Interaction	F (1, 11) = 3.564	P = 0.0857
	Treatment	F (1, 11) = 2.982	P = 0.1121
	Genotype	F (1, 11) = 2.415	P = 0.1484
EPL	Interaction	F (1, 11) = 1.257	P = 0.2862
	Treatment	F (1, 11) = 0.2280	P = 0.6423
	Genotype	F (1, 11) = 0.6497	P = 0.4373
MiL	Interaction	F (1, 11) = 1.440	P = 0.2553
	Treatment	F (1, 11) = 0.3073	P = 0.5904
	Genotype	F (1, 11) = 0.2831	P = 0.6052
IPL	Interaction	F (1, 11) = 2.797	P = 0.1226
	Treatment	F (1, 11) = 3.624	P = 0.0834
	Genotype	F (1, 11) = 5.789	P = 0.0349*
GrL	Interaction	F (1, 11) = 0.6114	P = 0.4508
	Treatment	F (1, 11) = 0.9532	P = 0.3499
	Genotype	F (1, 11) = 4.094	P = 0.0680
OB	Interaction	F (1, 11) = 0.7569	P = 0.4029
	Treatment	F (1, 11) = 3.088	P = 0.1067
	Genotype	F (1, 11) = 0.3181	P = 0.5841
AON	Interaction	F (1, 11) = 0.6770	P = 0.4281
	Treatment	F (1, 11) = 0.7626	P = 0.4012
	Genotype	F (1, 11) = 0.3808	P = 0.5497
Pir	Interaction	F (1, 11) = 0.2440	P = 0.6311
	Treatment	F (1, 11) = 1.104	P = 0.3158
	Genotype	F (1, 11) = 1.200	P = 0.2966

Table S6. Volume statistical data. Comparison of hemispheres in WT.

Area	Source of variation	F (DFn, DFd)	P value
GL	Interaction	F (3, 26) = 1.100	P = 0.3671
	Treatment	F (3, 26) = 3.082	P = 0.0448*
	Hemisphere	F (1, 26) = 0.002876	P = 0.9576
EPL	Interaction	F (3, 26) = 1.315	P = 0.2909
	Treatment	F (3, 26) = 0.6201	P = 0.6083
	Hemisphere	F (1, 26) = 0.008555	P = 0.9270
MiL	Interaction	F (3, 26) = 0.1182	P = 0.9486
	Treatment	F (3, 26) = 0.3088	P = 0.8188
	Hemisphere	F (1, 26) = 0.2898	P = 0.5949
IPL	Interaction	F (3, 26) = 0.4022	P = 0.7526
	Treatment	F (3, 26) = 0.7296	P = 0.5437
	Hemisphere	F (1, 26) = 1.361	P = 0.2540
GrL	Interaction	F (3, 26) = 1.285	P = 0.3004
	Treatment	F (3, 26) = 1.610	P = 0.2112
	Hemisphere	F (1, 26) = 0.7165	P = 0.4050
OB	Interaction	F (3, 26) = 2.146	P = 0.1187
	Treatment	F (3, 26) = 0.7113	P = 0.5541
	Hemisphere	F (1, 26) = 0.01958	P = 0.8898
AON	Interaction	F (3, 26) = 1.301	P = 0.2953
	Treatment	F (3, 26) = 3.114	P = 0.0434*
	Hemisphere	F (1, 26) = 1.421	P = 0.2440
Pir	Interaction	F (3, 26) = 0.02933	P = 0.9931
	Treatment	F (3, 26) = 4.771	P = 0.0088** (t)
	Hemisphere	F (1, 26) = 3.973	P = 0.0568

(t) WT saline 2 months–WT saline 4 months: t_6= 2.825; P = 0.0302.

Table S7. Volume statistical data. Comparison of hemispheres in TG.

Area	Source of variation	F (DFn, DFd)	P value
GL	Interaction	F (1, 10) = 0.1720	P = 0.6871
	Treatment	F (1, 10) = 0.1246	P = 0.7314
	Hemisphere	F (1, 10) = 0.008586	P = 0.9280
EPL	Interaction	F (1, 10) = 0.1370	P = 0.7190
	Treatment	F (1, 10) = 0.7583	P = 0.4043
	Hemisphere	F (1, 10) = 0.0001406	P = 0.9908
MiL	Interaction	F (1, 10) = 0.7184	P = 0.4165
	Treatment	F (1, 10) = 4.704	P = 0.0553
	Hemisphere	F (1, 10) = 0.6085	P = 0.4534
IPL	Interaction	F (1, 10) = 0.8659	P = 0.3740
	Treatment	F (1, 10) = 5.011	P = 0.0491*
	Hemisphere	F (1, 10) = 1.998	P = 0.1879
GrL	Interaction	F (1, 10) = 0.2270	P = 0.6440
	Treatment	F (1, 10) = 0.1053	P = 0.7522
	Hemisphere	F (1, 10) = 0.09657	P = 0.7624
OB	Interaction	F (1, 10) = 0.6403	P = 0.4422
	Treatment	F (1, 10) = 6.183	P = 0.0322*
	Hemisphere	F (1, 10) = 2.735	P = 0.1292
AON	Interaction	F (1, 10) = 3.401	P = 0.0950
	Treatment	F (1, 10) = 0.01606	P = 0.9017
	Hemisphere	F (1, 10) = 2.715	P = 0.1304
Pir	Interaction	F (1, 10) = 0.4561	P = 0.5147
	Treatment	F (1, 10) = 1.092	P = 0.3207
	Hemisphere	F (1, 10) = 0.2395	P = 0.6351

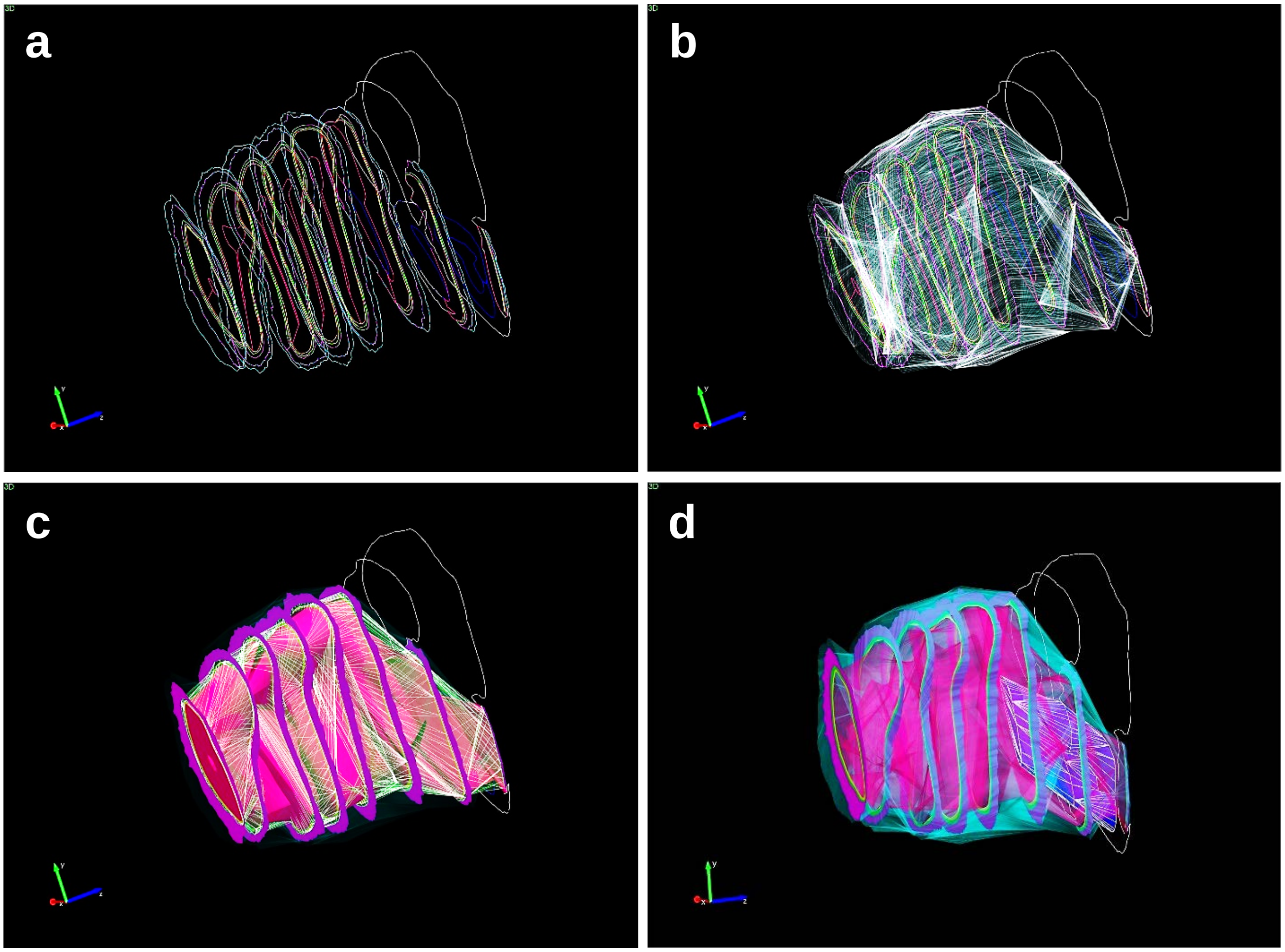

Fig. S5 OB and AON volume 3D model. OB and AON volume 3D model. OB layers (GL: blue, EPL: violet, MiL: green, IPL: yellow and GrL: rose) and AON (dark blue) boundaries were traced using Stereo Investigator software (**a**). GL was marked by "blue shell surface" (**b**). EPL and IPL were marked by "flat surface" and MiL and GrL showed "shell appearance" (**c**). OB and AON 3D model (**d**). For abbreviations, see list.
