# Supplementary material for: Neurodegeneration and contralateral α-synuclein induction after intracerebral α-synuclein injections in the anterior olfactory nucleus of a Parkinson’s disease A53T mouse model
Source: Acta Neuropathol Commun. 2019 Apr 15;7:56. doi: 10.1186/s40478-019-0713-7 (PMC6463651; doi:10.1186/s40478-019-0713-7)
Supplement: Supplementary file 4 — Stereological α-synuclein, NeuN, Iba-1 and GFAP quantification. Table S8. Statistical data of stereological α-synuclein quantification. Table S9. Statistical data of NeuN quantification (Mann-Whitney test). Table S10. Statistical data of stereological Iba-1 quantification. Comparison of genotype in the right hemisphere. Table S11. Statistical data of stereological Iba-1 quantification. Comparison of genotype in the left hemisphere. Table S12. Statistical data of stereological Iba-1 quantification. Comparison of hemispheres in WT. Table S13. Statistical data of stereological Iba-1 quantification. Comparison of hemispheres in TG. Table S14. Statistical data of GFAP quantification. Comparison of genotype in the right hemisphere. Table S15. Statistical data of GFAP quantification. Comparison of genotype in the left hemisphere. Table S16. Statistical data of GFAP quantification. Comparison of hemispheres in WT. Table S17. Statistical data of GFAP quantification. Comparison of hemispheres in TG. Figure S6. AON injection site labeled with different markers. Figure S7. α-synucleinopathy in caudate-putamen. Figure S8. α-synucleinopathy in substantia nigra. (PDF 814 kb) [file 40478_2019_713_MOESM4_ESM.pdf]

ADDITIONAL FILE 4

Neurodegeneration and contralateral α-synuclein induction after intracerebral α-synuclein injections in the anterior olfactory nucleus of a Parkinson's disease A53T mouse model

Alicia Flores-Cuadrado[1], Daniel Saiz-Sanchez[1], Alicia Mohedano-Moriano[2], Alino Martinez-Marcos[1], Isabel Ubeda-Bañon[1]*

[1]Neuroplasticity and Neurodegeneration Laboratory, CRIB, Ciudad Real Medical School, University of Castilla-La Mancha, Ciudad Real, Spain.
[2]School of Occupational Therapy, Speech Therapy and Nursing, University of Castilla-La Mancha, Talavera de la Reina, Spain.

Alicia.flores@uclm.es
Daniel.saiz@uclm.es
Alicia.mohedano@uclm.es
Alino.martinez@uclm.es

Address for correspondence:
Isabel Ubeda-Bañon
University of Castilla-La Mancha
Ciudad Real Medical School
Camino de Moledores s/n
13071 Ciudad Real (Spain)
Phone: 926295300 6835
E-mail Isabel.ubeda@uclm.es

Stereological α-synuclein, NeuN, Iba-1, GFAP quantification

Table S8. Statistical data of stereological α-synuclein quantification.

Area	Group (TG mice)	Comparisons (cells/mm^3)	t-test	P value
OB	Saline-injection	Left hemisphere × Right hemisphere	$t_6 = 6.948$	P = 0.0004***
	α-syn-injection	Left hemisphere × Right hemisphere	$t_4 = 1.605$	P = 0.1838
	α-syn-injection x Saline-injection	Right hemisphere × Right hemisphere	$t_5 = 2.080$	P = 0.0921
	α-syn-injection x Saline-injection	Left hemisphere × Left hemisphere	$t_5 = 3.805$	P = 0.0126*
AON	Saline-injection	Left hemisphere × Right hemisphere	$t_6 = 3.197$	P = 0.0187*
	α-syn-injection	Left hemisphere × Right hemisphere	$t_4 = 1.315$	P = 0.2588
	α-syn-injection x Saline-injection	Right hemisphere × Right hemisphere	$t_5 = 2.310$	P = 0.0689
	α-syn-injection x Saline-injection	Left hemisphere × Left hemisphere	$t_5 = 2.104$	P = 0.0893
Pir	Saline-injection	Left hemisphere × Right hemisphere	$t_6 = 0.334$	P = 0.7493
	α-syn-injection	Left hemisphere × Right hemisphere	$t_4 = 0.115$	P = 0.9136
	α-syn-injection x Saline-injection	Right hemisphere × Right hemisphere	$t_5 = 2.225$	P = 0.0767
	α-syn-injection x Saline-injection	Left hemisphere × Left hemisphere	$t_5 = 2.514$	P = 0.0536

Table S9. Statistical data of NeuN quantification (Mann-Whitney test).

Area	Source of variation	P value
OB	WT-saline-RH x TG-saline-RH	P = 0.0286*
	WT-saline-LH x TG-saline-LH	P = 0.0286*
	WT-α-RH x TG-α-RH	P = 0.2286
	WT-α-LH x TG-α-LH	P = 0.1143
	TG-saline-RH x TG-α-RH	P = 0.0571
	TG-saline-LH x TG-α-LH	P = 0.0571
	WT-saline-RH x WT-α-RH	P = 0.4857
	WT-saline-LH x WT-α-LH	P = 0.1143
AON	WT-saline-RH x TG-saline-RH	P = 0.0286*
	WT-saline-LH x TG-saline-LH	P = 0.0286*
	WT-α-RH x TG-α-RH	P = 0.4000
	WT-α-LH x TG-α-LH	P = 0.0571
	TG-saline-RH x TG-α-RH	P = 0.0571
	TG-saline-LH x TG-α-LH	P = 0.0571
	WT-saline-RH x WT-α-RH	P = 0.4857
	WT-saline-LH x WT-α-LH	P = 0.8286
Pir	WT-saline-RH x TG-saline-RH	P = 0.0286*
	WT-saline-LH x TG-saline-LH	P = 0.0286*
	WT-α-RH x TG-α-RH	P > 0.9999
	WT-α-LH x TG-α-LH	P = 0.2286
	TG-saline-RH x TG-α-RH	P = 0.0571
	TG-saline-LH x TG-α-LH	P = 0.0571
	WT-saline-RH x WT-α-RH	P = 0.0286*
	WT-saline-LH x WT-α-LH	P > 0.9999

Table S10. Statistical data of stereological Iba-1 quantification. Comparison of genotype in the right hemisphere.

Area	Source of variation	F (DFn, DFd)	P value
GL	Interaction	F (1, 11) = 0.04697	P = 0.8324
	Treatment	F (1, 11) = 1.256	P = 0.2863
	Genotype	F (1, 11) = 0.3596	P = 0.5609
EPL	Interaction	F (1, 11) = 0.06955	P = 0.7969
	Treatment	F (1, 11) = 1.053	P = 0.3268
	Genotype	F (1, 11) = 1.448	P = 0.2541
MiL	Interaction	F (1, 11) = 4.943	P = 0.0481* (t)
	Treatment	F (1, 11) = 0.8695	P = 0.3711
	Genotype	F (1, 11) = 4.519	P = 0.0570
IPL	Interaction	F (1, 11) = 0.3797	P = 0.5503
	Treatment	F (1, 11) = 1.604	P = 0.2315
	Genotype	F (1, 11) = 0.02487	P = 0.8775
GrL	Interaction	F (1, 11) = 1.013	P = 0.3358
	Treatment	F (1, 11) = 3.076	P = 0.1072
	Genotype	F (1, 11) = 0.1085	P = 0.7480
AON	Interaction	F (1, 11) = 0.03820	P = 0.8486
	Treatment	F (1, 11) = 0.01318	P = 0.9107
	Genotype	F (1, 11) = 1.427	P = 0.2573
Pir	Interaction	F (1, 11) = 0.3274	P = 0.5787
	Treatment	F (1, 11) = 1.016	P = 0.3351
	Genotype	F (1, 11) = 0.03855	P = 0.8479

(t) TG-α-right x TG-S-right: $t_5 = 3.410$; P = 0.0190.

Table S11. Statistical data of stereological Iba-1 quantification. Comparison of genotype in the left hemisphere.

Area	Source of variation	F (DFn, DFd)	P value
GL	Interaction	F (1, 11) = 3.982	P = 0.0714
	Treatment	F (1, 11) = 0.1076	P = 0.7490
	Genotype	F (1, 11) = 0.05624	P = 0.8169
EPL	Interaction	F (1, 11) = 0.1320	P = 0.7233
	Treatment	F (1, 11) = 2.039	P = 0.1811
	Genotype	F (1, 11) = 1.927	P = 0.1925
MiL	Interaction	F (1, 11) = 3.576	P = 0.0852 (t1)
	Treatment	F (1, 11) = 1.189	P = 0.2989
	Genotype	F (1, 11) = 0.7315	P = 0.4106
IPL	Interaction	F (1, 11) = 4.346e-005	P = 0.9949
	Treatment	F (1, 11) = 2.225	P = 0.1639
	Genotype	F (1, 11) = 0.2819	P = 0.6060
GrL	Interaction	F (1, 11) = 2.116	P = 0.1737
	Treatment	F (1, 11) = 0.004102	P = 0.9501
	Genotype	F (1, 11) = 0.3776	P = 0.5514
AON	Interaction	F (1, 11) = 0.7252	P = 0.4126
	Treatment	F (1, 11) = 0.2762	P = 0.6096
	Genotype	F (1, 11) = 0.002602	P = 0.9602
Pir	Interaction	F (1, 11) = 0.4845	P = 0.5008
	Treatment	F (1, 11) = 0.6570	P = 0.4348 (t2)
	Genotype	F (1, 11) = 0.05178	P = 0.8242

(t1) TG-α-left × TG-S-left: $t_5 = 3.626$; P = 0.0151.

(t2) TG-α-left × TG-S-left: $t_5 = 2.620$; P = 0.0471.

Table S12. Statistical data of stereological Iba-1 quantification. Comparison of hemispheres in WT.

Area	Source of variation	F (DFn, DFd)	P value
GL	Interaction	F (3, 26) = 1.253	P = 0.3108
	Treatment × Time	F (3, 26) = 52.42	P < 0.0001****
	Hemisphere	F (1, 26) = 0.01173	P = 0.9146
EPL	Interaction	F (3, 26) = 1.470	P = 0.2456
	Treatment × Time	F (3, 26) = 82.29	P < 0.0001****
	Hemisphere	F (1, 26) = 0.2745	P = 0.6047
MiL	Interaction	F (3, 26) = 0.1273	P = 0.9431
	Treatment × Time	F (3, 26) = 39.19	P < 0.0001****
	Hemisphere	F (1, 26) = 0.1012	P = 0.7530
IPL	Interaction	F (3, 26) = 0.1904	P = 0.9020
	Treatment × Time	F (3, 26) = 36.83	P < 0.0001****
	Hemisphere	F (1, 26) = 0.05854	P = 0.8107
GrL	Interaction	F (3, 26) = 1.304	P = 0.2944
	Treatment × Time	F (3, 26) = 149.7	P < 0.0001****
	Hemisphere	F (1, 26) = 1.179	P = 0.2876
AON	Interaction	F (3, 26) = 1.304	P = 0.2944
	Treatment × Time	F (3, 26) = 149.7	P < 0.0001****
	Hemisphere	F (1, 26) = 1.179	P = 0.2876
Pir	Interaction	F (3, 26) = 0.06495	P = 0.9779
	Treatment × Time	F (3, 26) = 35.75	P < 0.0001****
	Hemisphere	F (1, 26) = 0.001407	P = 0.9704

Table S13. Statistical data of stereological Iba-1 quantification. Comparison of hemispheres in TG.

Area	Source of variation	F (DFn, DFd)	P value
GL	Interaction	F (1, 10) = 0.1615	P = 0.6962
	Treatment	F (1, 10) = 2.842	P = 0.1227
	Hemisphere	F (1, 10) = 0.3787	P = 0.5520
EPL	Interaction	F (1, 10) = 1.320	P = 0.2774
	Treatment	F (1, 10) = 0.006174	P = 0.9389
	Hemisphere	F (1, 10) = 0.9160	P = 0.3611
MiL	Interaction	F (1, 10) = 0.1243	P = 0.7318
	Treatment	F (1, 10) = 24.35	P = 0.0006***
	Hemisphere	F (1, 10) = 3.098	P = 0.1089
IPL	Interaction	F (1, 10) = 0.08663	P = 0.7745
	Treatment	F (1, 10) = 1.997	P = 0.1880
	Hemisphere	F (1, 10) = 0.06282	P = 0.8072
GrL	Interaction	F (1, 10) = 0.3451	P = 0.5699
	Treatment	F (1, 10) = 3.261	P = 0.1011
	Hemisphere	F (1, 10) = 0.001080	P = 0.9744
AON	Interaction	F (1, 10) = 0.1234	P = 0.7326
	Treatment	F (1, 10) = 0.002316	P = 0.9626
	Hemisphere	F (1, 10) = 0.2034	P = 0.6616
Pir	Interaction	F (1, 10) = 7.081	P = 0.0239* (t)
	Treatment	F (1, 10) = 0.007362	P = 0.9333
	Hemisphere	F (1, 10) = 0.07785	P = 0.7859

(t) TG-α-left × TG-S-left: t_5 = 2.620; P = 0.0471.

Table S14. Statistical data of GFAP quantification. Comparison of genotype in the right hemisphere.

Area	Source of variation	F (DFn, DFd)	P value
OB	Interaction	F (1, 11) = 0.2590	P = 0.6208
	Treatment	F (1, 11) = 0.6954	P = 0.4221
	Genotype	F (1, 11) = 32.13	P = 0.0001***
AON	Interaction	F (1, 11) = 1.121	P = 0.3123
	Treatment	F (1, 11) = 3.740	P = 0.0793
	Genotype	F (1, 11) = 11.73	P = 0.0057** (t)
Pir	Interaction	F (1, 11) = 0.3859	P = 0.5471
	Treatment	F (1, 11) = 0.3573	P = 0.5621
	Genotype	F (1, 11) = 3.034	P = 0.1094

(t) WT saline 2m-TG saline 2m: t_6 = 3.134; P = 0.0202.

Table S15. Statistical data of GFAP quantification. Comparison of genotype in the left hemisphere.

Area	Source of variation	F (DFn, DFd)	P value
OB	Interaction	F (1, 11) = 0.9921	P = 0.3406
	Treatment	F (1, 11) = 0.08698	P = 0.7735
	Genotype	F (1, 11) = 22.10	P = 0.0006***
AON	Interaction	F (1, 11) = 0.8663	P = 0.3720
	Treatment	F (1, 11) = 0.03401	P = 0.8570
	Genotype	F (1, 11) = 30.14	P = 0.0002***
Pir	Interaction	F (1, 11) = 1.283	P = 0.2814
	Treatment	F (1, 11) = 0.4568	P = 0.5131
	Genotype	F (1, 11) = 1.287	P = 0.2807

Table S16. Statistical data of GFAP quantification. Comparison of hemispheres in WT.

Area	Source of variation	F (DFn, DFd)	P value
OB	Interaction	F (3, 26) = 0.2841	P = 0.8364
	Treatment	F (3, 26) = 4.233	P = 0.0146*
	Hemisphere	F (1, 26) = 1.315	P = 0.2619
AON	Interaction	F (3, 26) = 0.05543	P = 0.9824
	Treatment	F (3, 26) = 2.106	P = 0.1239
	Hemisphere	F (1, 26) = 0.5590	P = 0.4614
Pir	Interaction	F (3, 26) = 1.279	P = 0.3024
	Treatment	F (3, 26) = 2.273	P = 0.1038
	Hemisphere	F (1, 26) = 0.6240	P = 0.4367

Table S17. Statistical data of GFAP quantification. Comparison of hemispheres in TG.

Area	Source of variation	F (DFn, DFd)	P value
OB	Interaction	F (1, 10) = 0.9984	P = 0.3413
	Treatment	F (1, 10) = 0.009101	P = 0.9259
	Hemisphere	F (1, 10) = 0.6813	P = 0.4284
AON	Interaction	F (1, 10) = 3.763	P = 0.0811
	Treatment	F (1, 10) = 2.085	P = 0.1793
	Hemisphere	F (1, 10) = 0.5549	P = 0.4735
Pir	Interaction	F (1, 10) = 0.03984	P = 0.8458
	Treatment	F (1, 10) = 1.002	P = 0.3405
	Hemisphere	F (1, 10) = 0.05028	P = 0.8271

1.1 Supplementary Figures

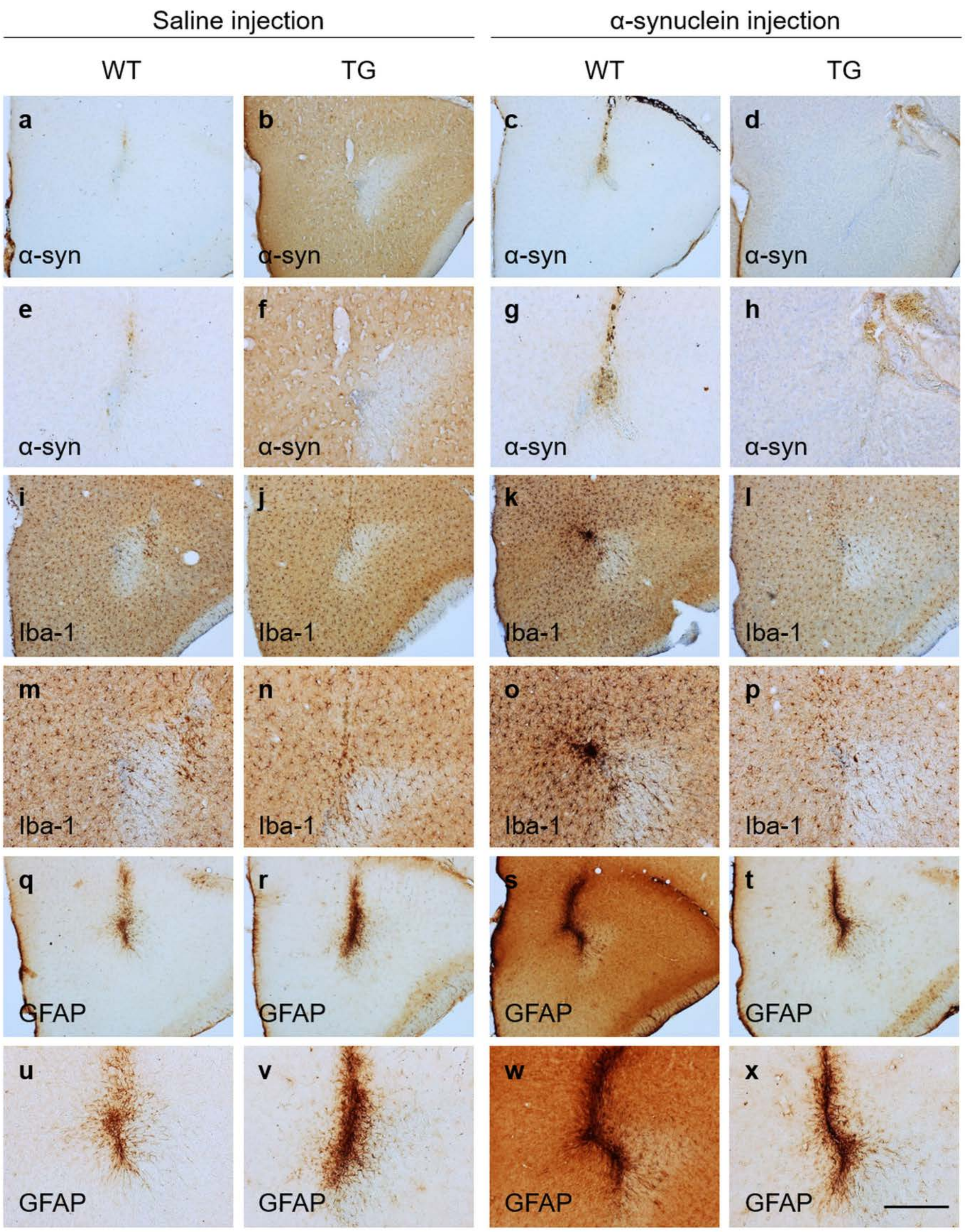

Fig. S6 AON injection site labeled with different markers. α-synuclein (**a-h**), Iba-1 (**i-p**) and GFAP (**q-x**). Scale bars: **a-d**, **i-l**, **q-t**, 500 μm; **e-h**, **m-p**, **u-x** 250 μm. For abbreviations, see list.

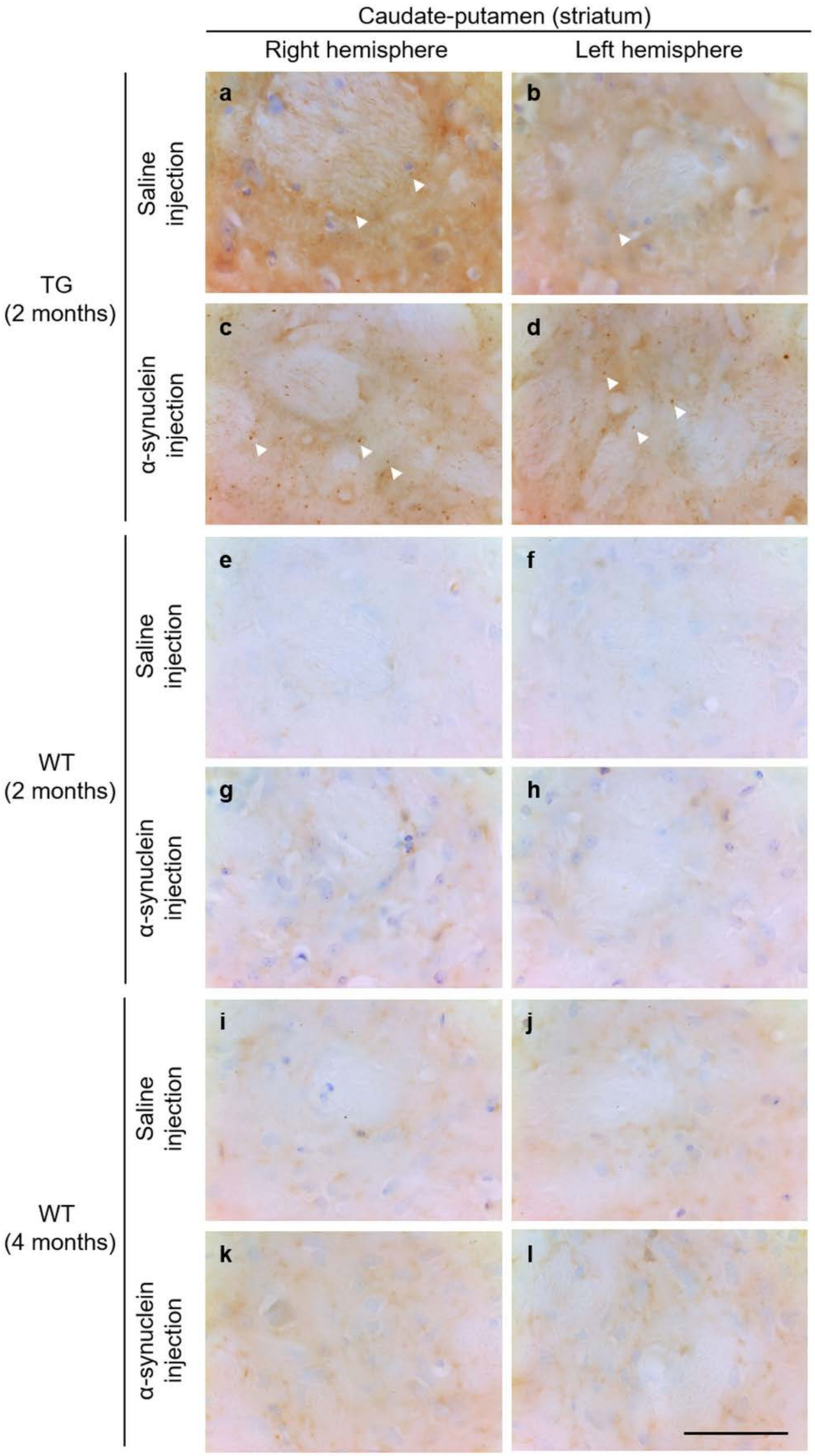

Fig. S7 α-synucleinopathy in the caudate-putamen (**a**-**l**). Arrows indicate α-synuclein aggregates around striosomes (organization of afferent and efferent fibers in the striatum) (**a**-**d**). These aggregates were not found in saline-injected and α-synuclein-injected WT animals (**e**-**l**). Scale bars: 50 μm.

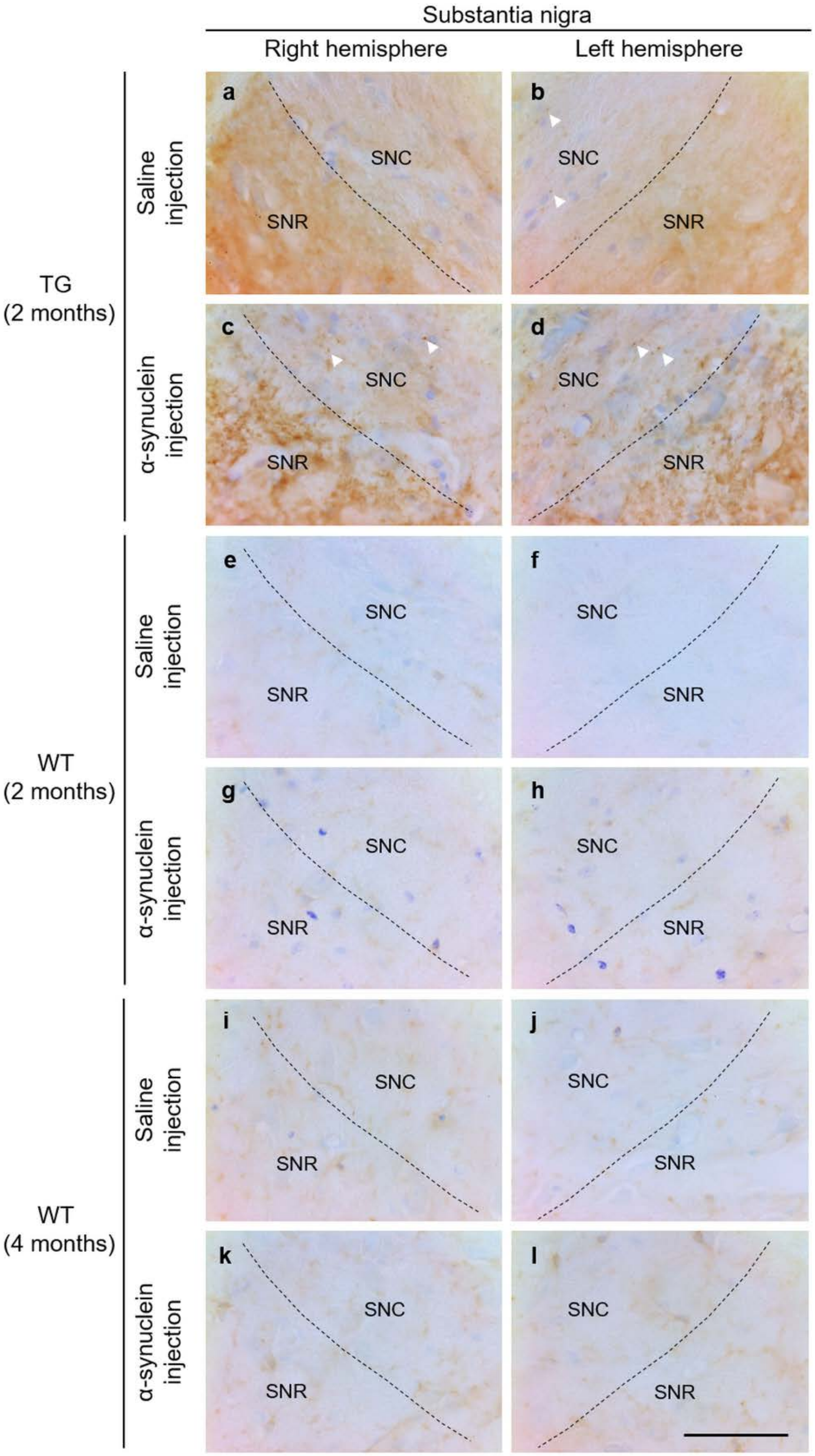

Fig. S8 α-synucleinopathy in the substantia nigra (**a**-**l**). Arrows indicate α-synuclein aggregates, mainly in substantia nigra compact part (SNC) (**a**-**d**). These aggregates were not observed in saline-injected and α-synuclein-injected WT animals (**e**-**l**). Scale bars: 50 μm.
